# Dual ENPP1/ATM depletion blunts DNA damage repair boosting radioimmune efficacy to abrogate triple-negative breast cancer

**DOI:** 10.1038/s41392-025-02271-2

**Published:** 2025-06-13

**Authors:** Borja Ruiz-Fernández de Córdoba, Karmele Valencia, Connor Welch, Haritz Moreno, Susana Martínez-Canarias, Carolina Zandueta, Eduardo Gómez, Alfonso Calvo, Nerea Otegui, Mirari Echepare, Ignacio Garzón, Daniel Ajona, David Lara-Astiaso, Elisabeth Guruceaga, Laura Guembe, Rubén Pío, Ignacio Melero, Silve Vicent, Fernando Pastor, Rafael Martínez-Monge, Fernando Lecanda

**Affiliations:** 1 Solid Tumors Program. Division of Oncology, Center for Applied Medical Research (CIMA)-University of Navarra, Pamplona, Spain; 2 IdiSNA, Navarra Institute for Health Research, Pamplona, Spain; 3 Consorcio de Investigación Biomédica en Red de Cáncer (CIBERONC), Madrid, Spain; 4 Molecular Therapeutics and Innovation Therapeutics Programs, CIMA-University of Navarra, Pamplona, Spain; 5 Department of Pathology, Anatomy and Physiology, School of Medicine, University of Navarra, Pamplona, Spain; 6 Department of Haemato-oncology, University of Cambridge, Cambridge, UK; 7 Wellcome Trust-Medical Research Council, Cambridge Stem Cell Institute, Cambridge, UK; 8 Bioinformatics Core Facility, CIMA-University of Navarra, Pamplona, Spain; 9 Morphology Core Facility, CIMA-University of Navarra, Pamplona, Spain; 10 Department of Biochemistry and Genetics, School of Sciences, University of Navarra, Pamplona, Spain; 11 Immunology and Immunotherapy, CIMA-University of Navarra and Cancer Center Clínica Universidad de Navarra (CCUN), Pamplona, Spain; 12 Oncology, Clínica, University of Navarra, CCUN, Pamplona, Spain; 13 Radiation Oncology, Clínica University of Navarra, CCUN, Pamplona, Spain; 14 Surgery, Clínica University of Navarra, CCUN, Pamplona, Spain

**Keywords:** Breast cancer, Metastasis, Preclinical research, Molecular medicine, Target identification

## Abstract

The ATP-hydrolytic ectoenzyme ENPP1 has been implicated in the metastasis and recurrence in triple-negative breast cancer (TNBC), primarily by contributing to tumor cell survival and treatment resistance. However, the precise mechanisms remain unclear. In a model of local recurrence (LR), circulating tumor cells (CTC) engrafting in the post-resection tumor bed developed a radioresistant phenotype linked to an ENPP1^+^-gene signature which was also identified in TNBC patients, suggesting ENPP1´s role in genome integrity. Blockade of ENPP1 using a permeable ENPP1 inhibitor (AVA-NP-695) reduced radioresistance, mechanistically attributed to decreased homologous recombination (HR) resulting in persistent DNA damage, as evidenced by enhanced tail moment and sustained γH2AX formation. This impaired DNA damage repair (DDR) sensitized tumor cells to ionizing radiation (IR). Notably, several DDR inhibitors (i) (including PARPi and ATMi) showed the highest synergy score in a targeted pharmacological screening. In vivo, dual ENPP1/ATM inhibition heightened radiosensitivity, compromised tumor cell survival and enhanced STING-TBK1 signaling by preventing ENPP1-mediated cGAMP hydrolysis. This resulted in robust innate and long-lasting adaptive antitumor immune memory responses, leading to significant tumor regression. Remarkably, combined treatment post-IR reduced spontaneous metastasis and local recurrence, and induced abscopal effects that impacted distant tumor spread in orthotopic tumor models. Thus, these findings position ENPP1 as a critical link between genome integrity and immunosuppression, offering promising translational opportunities for treating local or distant dissemination in TNBC.

## Introduction

Distant dissemination and local failure (LF) are common complications in a variety of solid tumors.^1^ In triple negative breast cancer (TNBC), 10–20% of patients present metastasis at diagnosis, with median event-free survival under a year. Among non-metastatic cases treated, over 40% fail to achieve a complete pathological response despite state-of-the-art treatments. These patients face high rates of local failure (LF) and distant metastases, resulting in a 5-year survival rate as low as 25%.^2^ Both scenarios pose a therapeutic conundrum and a vexing clinical challenge, as treatment options are limited and often associated with a bleak prognosis.

A poor understanding of local and distant dissemination post-treatment has been inferred from clinical observations.^3,4^ Residual tumor cells, circulating tumor cells (CTC), and cells that extravasate and settle in other organs manage to evade the limitations imposed by immunosurveillance and treatment effects, thereby contributing to tumor outgrowth both locally and at distant sites.^5,6^ During tumor progression, tumor-released factors are established at distant sites, with a complex milieu involving extracellular remodeling, the mobilization of bone-marrow derived cells, and other cellular components preparing the so-called pre-metastatic niche.^7,8^ Similarly, these factors could establish a tumor-primed environment together with wound healing and inflammation perpetuated throughout tumor growth favoring local relapse.^9^ Defining the critical mechanisms of resilience that enable tumor cell survival amidst various insults may uncover novel vulnerabilities.

Recent studies have revealed the emerging roles of ENPP1 (ectonucleotide pyrophosphatase phosphodiesterase 1, or CD203a), a key transmembrane purinergic regulator, in mediating distant dissemination and local recurrence in tumors.^10,11^ ENPP1 is frequently overexpressed in metastatic tumors across many solid neoplasms.^10^ Its expression confers a prometastatic phenotype and contributes to resistance against immune checkpoint blockade (ICB).^10^ In TNBC, ENPP1 fosters a strong immunosuppressive tumor microenvironment with patients exhibiting elevated ENPP1 levels experiencing shorter recurrence-free survival.^12^ TNBC is characterized by high chromosomal instability (CIN), a hallmark present in 60–80% of tumors, which leads to the formation of micronuclei containing fragmented DNA that can be recognized by DNA sensor machinery triggering STING (Stimulator of Interferon Genes) activation. ENPP1 hydrolyzes cGAMP and antagonizes the activation of STING thereby promoting evasion from immunosurveillance.^13–15^ Furthermore, ENPP1 is overexpressed in recurrent breast cancer tumors. Blockade of ENPP1 in combination with fractionated dose (FD) radiotherapy effectively reverses immunosuppression and reduces LF in preclinical models.^12^

However, the mechanistic underpinning of how ENPP1 in tumor cells enables an enhanced resilience to the treatment challenges at local and distant sites remains poorly understood. Characterization of this aspect may reveal signaling pathways supporting treatment resistance that expose novel targets for therapeutic exploitation.

In this study, we identified a novel ENPP1^+^-invigorated phenotype of radioresistance that entailed enhanced proficiency to DNA damage repair. Genetic or pharmacological ENPP1 blockade impaired DDR by lowering HR, reverted this phenotype and synergized with currently preclinically investigated DDR inhibitors increasing radiosensitivity and enhancing antitumor immune responses both in models of local and distant dissemination. Our results uncover novel mechanistic vulnerabilities that enhance antitumor therapeutic effectiveness, with implications in preventing local and distant spread, and present promising translational opportunities for the treatment of patients with TNBC.

## Results

### Early engrafted CTC-in acquire an enhanced DNA integrity signature

We postulated that the acquisition of an ENPP1^+^ phenotype would endow cells with an enhanced competency to overcome treatment challenges.

**Fig. 1:**
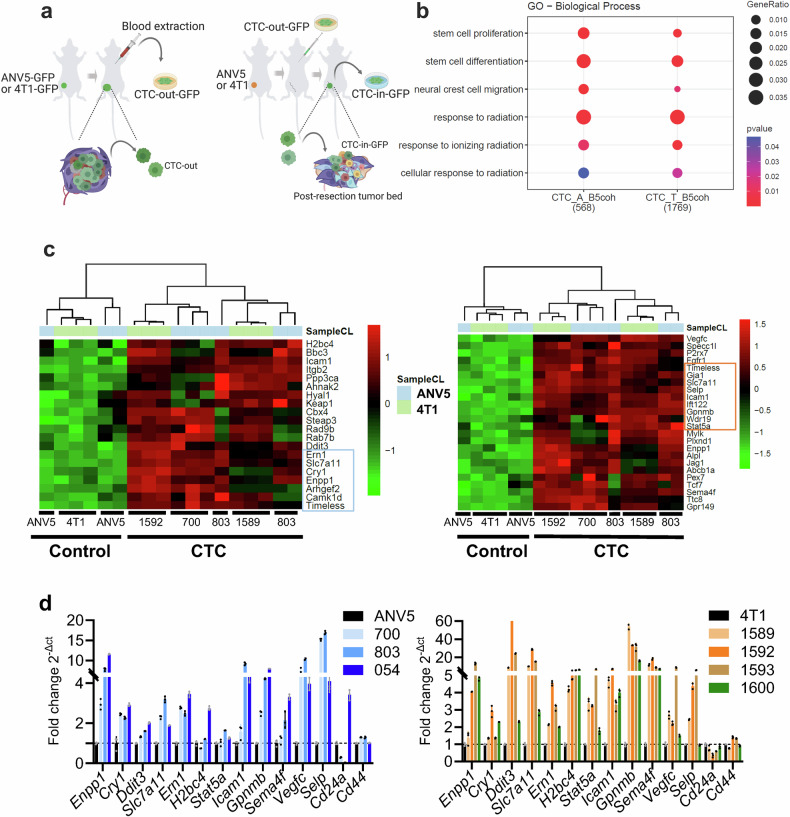
CTC acquire an ENPP1^+^ signature displaying genome integrity and stemness traits. **a** Schematic of the isolation of engrafting “CTC-in” at the RTB after the intracardiac inoculation in previously orthotopically tumor resected mice (generated by BioRender). **b** Selected hierarchical functional GO categories based on their biological relevance related to the observed phenotype, obtained by transcriptomic analysis of gene signatures from two different independently isolated CTC-in cells derived from each ANV5 (CTC_A_B5coh) and 4T1 (CTC_T_B5coh) cell lines, compared to their respective parental cells using RNA-seq. Number of coherent genes with B > 5 appears in parenthesis for each cell line. **c** Hierarchical cluster of transcriptomic upregulated genes related to “Response to radiation” *(Left panel)* and Stemness *(Right panel)* GO categories obtained in two different independently isolated CTC-in cells derived from each ANV5 and 4T1 parental cell lines. **d** Validation by RT-qPCR of commonly regulated genes expressed in different CTC-in derivatives from ANV5 (700, 803, and 054) and 4T1 (1589, 1592, 1593 and 1600) isolated at the RTB compared to their respective parental ANV5 and 4T1 cell lines

We first investigated whether human circulating tumor cells (CTC) exhibited increased ENPP1 expression levels in breast cancer patients by utilizing ctcRbase. Isolated CTC showed increased ENPP1 levels as compared to primary tumors in one published dataset (GSE41245) but this finding was not consistent with others (Supplementary Fig. 1a). This may be explained by the fact that CTC in unselected patients may originate from productive local or distant sites, constituting a confounding factor. To overcome this hurdle, we took advantage of isolated CTC (CTC-in) reaching the tumor-preconditioned post-resection tumor bed (RTB) in two murine models of LF.^12^ Briefly, CTC are collected from blood of incipient orthotopically implanted ANV5 and 4T1-derived tumors transduced with GFP, which we called CTC-out-GFP. CTC-out-GFP are expanded ex vivo and inoculated in the left cardiac ventricle in a group of tumor-resected mice that were previously orthotopically inoculated with unlabeled ANV5 and 4T1 cells, respectively. GFP^+^ tumor cells were isolated soon after recurrence in the RTB and expanded ex vivo (CTC-in) (Fig. 1a). Isolated CTC-in subpopulations derived from ANV5 (700Cy1 and 803Cy1) and 4T1 (1589Cy1 and 1592Cy1) cells recapitulate early events of engraftment and show increased gene expression levels of ENPP1.^12^

Interestingly, RNA-seq analysis of the isolated CTC-in subpopulations revealed an ENPP1^+^ phenotype characterized by several significantly enriched Gene Ontology (GO) biological categories in genes differentially expressed with a significance threshold of B > 5 common to both tumor cell models. From these categories, we selected those that were compatible with the in vivo phenotypic traits observed in both models (Supplementary Fig. 1b), which include “Stemness”, “Response to Radiation”, “Tissue remodeling”, and “Regulation to inflammatory response”, among others (Fig. 1b). We identified differentially expressed genes belonging to these categories, including those that were upregulated (Fig. 1c) or downregulated (Supplementary Fig. 1c) which exhibit coherence between CTC. In addition to ENPP1, the CTC-in subpopulations also expressed TIMELESS, which interacts with PARP1^16^; STAT5a, which plays a role in homologous recombination (HR) DNA repair^17^; and ERN1(IRE1α), an unfolded protein response (UPR) sensor that contributes to DNA damage response (DDR)^18^(Fig. 1c). Other genes associated with stem-like traits, such as CD24a and NUDT21, were found to be downregulated (Supplementary Fig. 1c).^19^ We validated a subset of these genes in a panel of previously isolated CTC-in subpopulations (Fig. 1d). A certain interdependency among a small subset of genes was detected upon forced ENPP1 expression (Supplementary Fig. 1d). These findings indicate that engrafted CTC were endowed with a transcriptional gene signature indicative of radiation responsiveness and stemness traits, compatible with an enhanced endurance to genotoxic stress.

### ENPP1^+^ CTC show radioresistance associated with a proficient ENPP1-mediated DDR mechanism

**Fig. 2:**
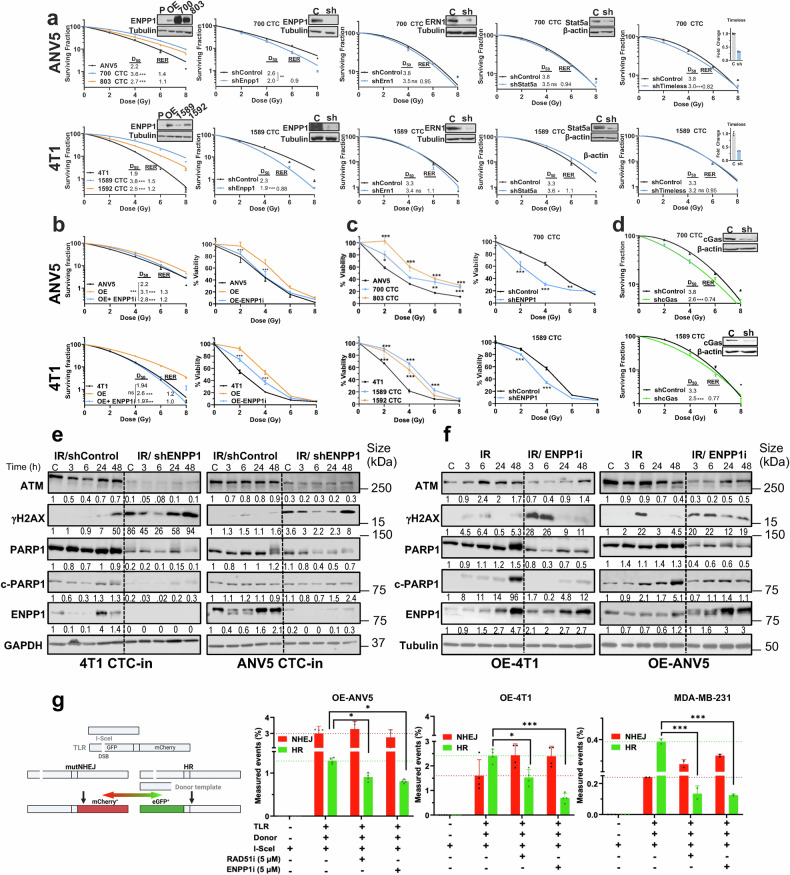
CTC-in cells display a radioresistant phenotype mediated by ENPP1. **a** Clonogenic assays with independently isolated CTC-in derived from ANV5 (700 and 803) and 4T1 (1589 and 1592) as well as CTC-in with silenced levels of ENPP1 using shRNAs targeting ENPP1, ERN1, TIMELESS, and STAT5a. The right inset depicts ENPP1 protein levels assessed by immunoblotting. An extra sum-of-squares F test was used for comparison. D_50_ and RER (Radiation enhancement ratio at 2 Gy) values are included. Ns Not significant, ** p < 0.01, ***p < 0.0001. **b** *Left panels:* Clonogenic assay performed in ENPP1-overexpressing cells (Top, OE-ANV5 and bottom, OE-4T1) alone or incubated with ENPP1i (5 µM for OE-ANV5 and 10 µM for OE-4T1) at the indicated doses of IR. An extra sum-of-squares F test was used for comparison. ***p < 0.0001. D_50_ and RER values are included. *Right panels:* Survival assay conducted with OE cells in similar conditions. One-way ANOVA was used for comparison of ENPP1i treatment with OE cells. **c** Survival assays using CTC-in derivatives (*Left*) or CTC-in cells with silenced levels of ENPP1 (Right). **d** Clonogenic assay of CTC-in with silenced levels of cGAS. Right inset depicts cGAS protein levels. Extra sum-of-squares F test was used for comparison, ***p < 0.0001. D_50_ and RER values are included. **e** Immunoblot for PARP1, cleaved-PARP1 (c-PARP1), ATM, phosphorylated H2AX (γH2AX), and ENPP1 in CTC-in cells derived from 4T1 and ANV5 with silenced ENPP1 levels (shENPP1) and shControl cells (transduced with shRNA Control) assessed at the indicated time points post-IR (2 Gy). Dashed lines segregate different treatments within individual immunoblots to enhance visualization. **f** Immunoblot evaluation for the indicated proteins in OE-ANV5 and OE-4T1 cell lysates after treatment with IR (2 Gy) or in combination with ENPP1i (5 µM). Bands of interest from representative immunoblots from three independent experiments are shown. **g** *Left panel:* Outline of the Traffic Light Reporter (TLR) assay (Modified from).^51^ Three plasmids are co-transduced (in gray): the TLR, the I-SceI encoding nuclease (I-SceI-T2A-IFP) and the Donor template (Donor-T2A-BFP). *Right panels:* Quantification of TLR readout after applying a nuclease titration gating analysis in cells co-transduced with I-SceI and Donor-T2A-BFP. RAD51i was used as a positive control for decreased HR. ENPP1i significantly reduced the percentage of normalized homologous recombination (HR) events in the OE cells and in human MDA-MB-231 cells, which endogenously express high levels of ENPP1. Average of 3 independent experiments. Mean ± SEM are represented. One-way ANOVA was used for comparison of HR events in treated cells against Control (TLR/Donor/SceI)

Based on the identification of a radiation resistance signature, we examined the functional endurance to IR-induced genotoxic insult of CTC-in derivatives from ANV5 (700 and 803) and 4T1 (1589 and 1592). These cells exhibit an enhanced radioresistance as compared to their respective parental cells in clonogenic assays (Fig. 2a). To discern the contribution of individual genes to this phenotype, we silenced ENPP1, STAT5a, ERN1, and TIMELESS in CTC-in derivatives. Silencing ENPP1 expression levels in these cell lines resulted in decreased radioresistance in both derivatives, while cells silenced for STAT5a, ERN1 and TIMELESS did not show consistent reductions in radioresistance compared to controls (Fig. 2a).

To further substantiate the role of ENPP1, we overexpressed ENPP1 (OE) in parental ANV5 or 4T1 cells. Interestingly, OE cells showed an increased radioresistance in clonogenic assays (Fig.2b). Interestingly, pharmacological abrogation of ENPP1 (ENPP1i) using the cell permeable inhibitor (AVA-NP-695) resulted in marked radiosensitivity, significantly diminishing the clonogenic activity and cell viability of the OE-treated cells (Fig. 2b). As anticipated, extracellular cGAMP levels were elevated following ENPP1i incubation in IR-treated OE-ANV5 cells (Supplementary Fig. 2a). Survival assays indicated increased viability in CTC-in derivatives (Fig. 2c, Supplementary Fig. 2b), an effect that was diminished in ENPP1-silenced cells (Fig. 2c).

Interestingly, in CTC-in derivatives, the silenced levels of cGAS, which catalyzes the synthesis of cGAMP, mirrored the shENPP1-mediated phenotype leading to a diminished radioresistance (Fig. 2d). Notably, CTC-in displayed similar tumor cell growth kinetics to their respective ANV5 and 4T1 parental cells (Supplementary Fig. 2c). CTC-in and other endogenously expressing ENPP1 human cells such as MCF-7 and MDA-MB-231 became radiosensitive after incubation with ENPP1i, whereas in non-transformed MCF10A cells, double IR and ENPPi treatment did not exhibit an additive effect compared to IR alone (Supplementary Fig. 2d). Of note, non-expressing ENPP1 parental cells (ANV5 and 4T1) were unaffected by ENPP1i treatment (Supplementary Fig. 2e). Thus, CTC-in acquired a radioresistant phenotype characterized by a multi-gene transcriptomic signature that involves the functional activity of ENPP1.

To further investigate the contribution of ENPP1 in radioresistance, we irradiated (2 Gy) CTC-in (shControl) and ENPP1-silenced cells (shENPP1) to induce DNA damage. Interestingly, IR induced sustained γH2AX levels indicative of unrepaired double strand breaks (DSB) over time, at earlier and higher levels in shENPP1 CTC-in (CTC-in derived from 4T1 and ANV5) compared to shControl cells, indicating a decreased ability to repair IR-induced DSB, which uncovers a novel vulnerability (Fig. 2e). γH2AX was undetected until 48 h post-IR in the 4T1-CTC-in control cells. However, IR induced a sustained increase at early time points that were enhanced at 48 h in both shENPP1 CTC-in cells (Fig. 2e). Additionally, IR-induced global levels of PARP1, which binds to DSB, were slightly attenuated in shENPP1 CTC-in cells (Fig. 2e), effects that were not mirrored at the transcriptional level (Supplementary Fig. 2f).

Based on the observation that tumors express varying levels of ENPP1,^12^ we sought to focus exclusively on the role mediated by ENPP1, avoiding the confounding effects from other components of the transcriptomic signature identified in CTC-in. Since these components could potentially contribute to the DDR mechanism, we irradiated parental cells with forced expression of ENPP1 (OE-ANV5 and OE-4T1) with and without incubation with ENPP1i. Consistent with previous findings, PARP1 levels were slightly attenuated in IR/ ENPP1i-treated cells compared to those treated with IR alone (Fig.2f).

Furthermore, IR (2 Gy) in OE-4T1 and OE-ANV5 cells did not induce DNA damage at baseline, but γH2AX was detected at 3 h and 6 h post-IR, respectively, indicating that ENPP1-OE cells display a proficient DNA damage repair (Fig. 2f). In contrast, ENPP1 blockade (ENPP1i) prevented the repair of IR-induced DSB, evidenced by sustained γH2AX detection as early as 3 h post-IR. Notably, ENPP1 levels increased post-IR in ENPP1i-treated cells. This elevation resulted from compensatory transcriptional mechanisms induced by ENPP1 blockade, along with decrease degradation of ENPP1, since actinomycin D did not totally block the increase in ENPP1 levels observed at 48 h (Supplementary Fig. 2g).

Since IR increases oxidative stress, we used a model that mimics this condition by incubating cells with H_2_O_2_ to assess nuclear ATM-phosphorylation and global PARylation, a post-translational modification affecting several components of the DDR machinery. Compared to ANV5, OE cells exhibited decreased PARylation levels upon incubation, which recovered much faster in ENPP1i-treated OE cells. Thus, ENPP1 influences the global levels of protein dePARylation (Supplementary Fig. 2h). Moreover, in ANV5 cells, nuclear phosphorylated-ATM levels in OE cells were detected as early as 30 min and were maintained at 60 min, but declined sharply in control and ENPP1i-treated cells at 60 min (Supplementary Fig. 2i). In contrast, in 4T1 cells, phosphorylated ATM levels remained low in OE cells compared to the sustained higher levels observed in control and ENPP1i-treated cells at 60 min. This indicates different phosphorylation kinetics among cell lines under these experimental conditions (Supplementary Fig. 2i). Collectively, these findings suggest that ENPP1 activity modulates post-translational modifications, affecting the activation kinetics of ATM in a cell-specific manner and conferring a proficient DDR phenotype that enables cells to overcome stress conditions.

To further investigate these findings, we assessed the mechanistic effects of ENPP1i on DNA repair by performing the traffic-light reporter (TLR) assay in cells with forced expression of ENPP1 (Fig. 2g). Briefly, cells were co-transduced with the TLR, the donor template, and a coding sequence for a SCE-1 endonuclease (I-SCE1) which generates DSB. At DSB sites, a prevailing competition occurs between homologous recombination (HR) and non-homologous end-joining (NHEJ), revealed by mCherry^+^ or GFP^+^ (indicative of NHEJ or HR, respectively). As expected, incubation with a RAD51i resulted in diminished HR capacity in both OE cell lines. Interestingly, incubation with ENPP1i also led to a marked decrease in the number of HR events indicating that ENPP1i impairs DDR through a mechanism involving HR inhibition. Similar results were observed in the human MDA-MB-231 cell line (Fig. 2g). Despite ENPP1 having modest impact in cell cycle residency, differences observed between the proportion of cells residing in non-HR (G0/G1) and HR competent (S/G2M) phases of the cell cycle upon ENPP1 blockade could not account for the marked decrease observed on HR assessed in the TLR assay (Supplementary Fig. 3a).

Collectively, these data suggest that ENPP1 contributes to DNA integrity, and its genetic or pharmacological abrogation cooperates with IR-induced DNA damage, leading to genome fragmentation that compromises the repair of DSB, ultimately diminishing tumor cell survival.

### Targeted pharmacological screen identifies synergy of ENPP1i with DDRi compounds

**Fig. 3:**
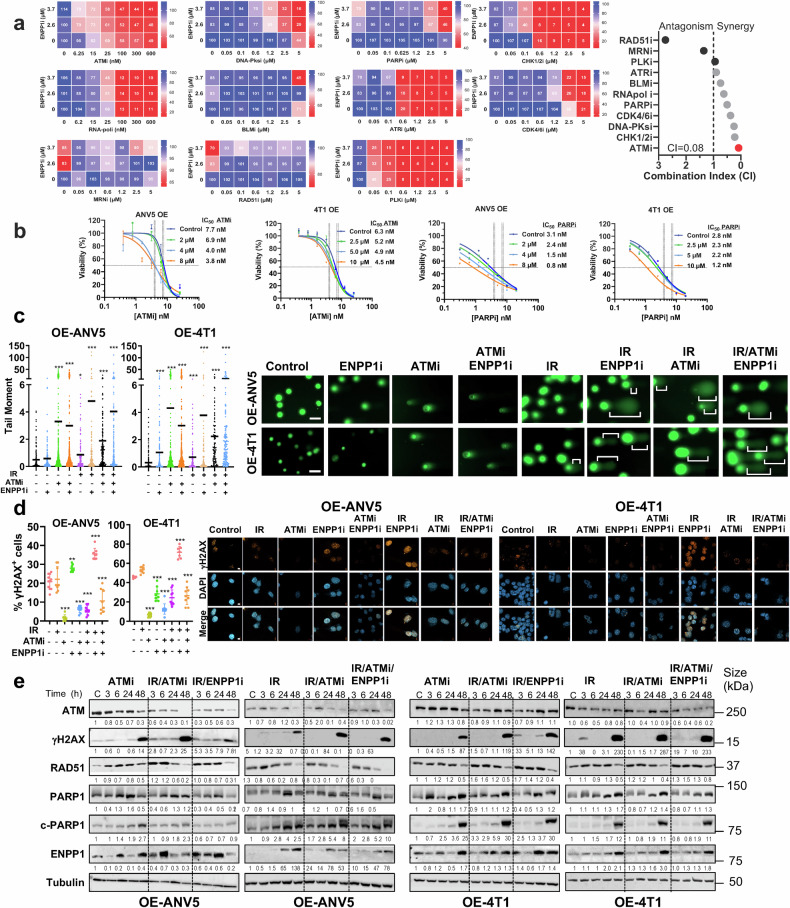
ENPP1 blockade synergizes with DDR inhibitors and boosts DNA damage post-IR. **a** *Left panels:* Heatmaps displaying the percentage of cell viability after 5 days of treatment with modulators of DNA integrity and ENPP1i from a targeted pharmacological screen. The x-axis features 11 kinase inhibitors that target components of the DNA damage response. *Right panel:* Synergy score. ATMi in combination with ENPP1i showed the highest synergistic effect (Combination Index close to 0). **b** Viability assay was conducted 5 days after incubation with ATMi or PARPi post-IR at increasing concentrations of ENPP1i showing a decrease in the IC_50_. **c** *Left panels:* Quantification of the Comet assay. *Right panels*: Representative images of the Comet assay showing the tail moment of the indicated cells treated with IR (2 Gy), ATMi (5 µM) and ENPP1i (5 µM). Brackets point to the tail length. Kruskal-Wallis was used for comparison and Dunn’s post-hoc multiple comparisons test against the Control group. ***P < 0.001. Scale bar = 50 µm. **d** *Left panels:* Quantitative assessment of the number of positively labeled cells with anti-γH2AX antibody performed by an in-house developed macro based on ImageJ®. Cells were incubated with ENPP1i (5 µM) and ATMi (5 µM) for 24 h. *n* > 100 cells were examined over three biologically independent experiments. Median and interquartile range are represented. One-way ANOVA was used for comparisons, and Dunnett´s multiple comparisons test against the Control group. *Right panels:* Representative immunofluorescence images of nuclear γH2AX (red) and nuclei (blue) in cells subjected to the indicated treatments. Scale bar = 10 µm. **e** Immunoblot analysis of protein expression levels of γHA2X, PARP1, cleaved PARP1 (c-PARP), ENPP1, RAD51, GAPDH, and Tubulin from cell lysates extracted from a time course after treatment with IR (2 Gy), IR/ENPP1i (5 µM), IR/ATMi (5 µM), or the triple combination in ANV5-OE and OE-4T1 cells. Normalization in each immunoblot was performed relative to the control cells (C) at time 0. Cross-comparison between immunoblots should take into account variation among control samples on each membrane. Dashed lines segregate different treatments within individual immunoblots to enhance visualization

Given the uncovered DNA repair vulnerability exposed by ENPP1, we sought to identify therapeutic hits with synergistic activity. To increase the translational value, we performed a targeted pharmacological screening using a compound library containing 11 inhibitors of key components of DDR or replication fork stability under clinical evaluation. Synergies occurred across several DNA damage compounds, including ATMi, CHK1/2i, PARPi, DNA-PKcsi, and CDK4/6i (Fig. 3a), whereas compounds targeting HR like RAD51, did not show such synergy. ATM, a key apical sensing DNA damage component initiating cellular responses to DSB repair, showed the highest synergy score (the lowest combination index, CI) with ENPP1i and was therefore chosen as a combinatorial partner to evaluate its therapeutic activity.^20^ Hence, increasing concentrations of ENPP1i diminished the IC_50_ of ATMi and PARPi in post-IR treated cells (Fig. 3b). Likewise, concurrent incubation of ATMi and ENPP1i in OE cell lines (Supplementary Fig. 3b) or in combination with IR showed a detrimental effect on cell growth kinetics and a concomitant apoptosis induction in murine and human cell lines endogenously expressing ENPP1(Supplementary Fig. 3c). However, the ENPP1 non-expressing cell line, MCF-10A, did not show a further decrease in cell viability (Supplementary Fig. 3c). Consistently, silenced ENPP1 levels in CTC-in lines showed an increased sensitivity to ATMi, PARPi and DNA-PKcsi (Supplementary Fig. 3d, e). These observations support the synergistic effects of dual treatment of ENPP1i with several DDRi and substantiate the role of ENPP1 promoting genome integrity.

Given the aforementioned findings, IR-induced DSB increased tail moments by comet assays, indicating increased DNA damage that nearly returned to baseline after 24 h, whereas incubation with ENPP1i after IR prevented DNA damage repair and significantly increased genome instability, as displayed by the enhanced percentage of tail moments detected (Fig. 3c). Furthermore, concomitant DNA damage was evidenced by the increased tail moment induced by ATMi when IR-induced conditions were further enhanced with additional ENPP1 blockade (Fig. 3c). Concurrently, immunofluorescence of γH2AX foci at 24 h post-IR showed a significant increase in unrepaired DSB upon incubation with ENPP1i (Fig. 3d). Despite the increased DNA damage noted in the comet assay at 24 h, a decrease in nuclear γH2AX foci was observed in IR-treated cells with ATMi at 24 h. Since ATM is an apical sensor of DNA damage that phosphorylates H2AX, phosphorylation of H2AX by ATM was blocked by ATMi, with levels remaining unchanged after the addition of ENPP1i compared to control cells (Fig. 3d). Thus, in cells subjected only to ATMi or ATMi after IR, we detected low levels of γH2AX foci at 24 h, despite the enhanced DNA damage observed in the comet assay. This low percentage of γH2AX foci was also observed at early time points (Supplementary Fig. 3f). Remarkably, levels of RAD51, which is crucial for HR, were downregulated at 48 h, suggesting that, rather than HR, NHEJ repair mechanisms were predominant, consistent with previous findings (Fig. 3e). Under these conditions, an increase in c-PARP1 was observed as early as 3 h was evidenced following IR and treatment combinations, declining at 48 h for OE-ANV5 cells. In contrast, strong increase was observed at 48 h in all treatments for 4T1 cells, indicating cell-specific kinetics of c-PARP1. Collectively, these data suggest that in ENPP1 expressing cells, ENPP1i/ATMi post-IR leads to unrepaired DSB which associates with impaired tumor cell growth kinetics in vitro.

### ENNP1i/ATMi post-IR abrogates tumor growth, boosts local control, and triggers abscopal effects

**Fig. 4:**
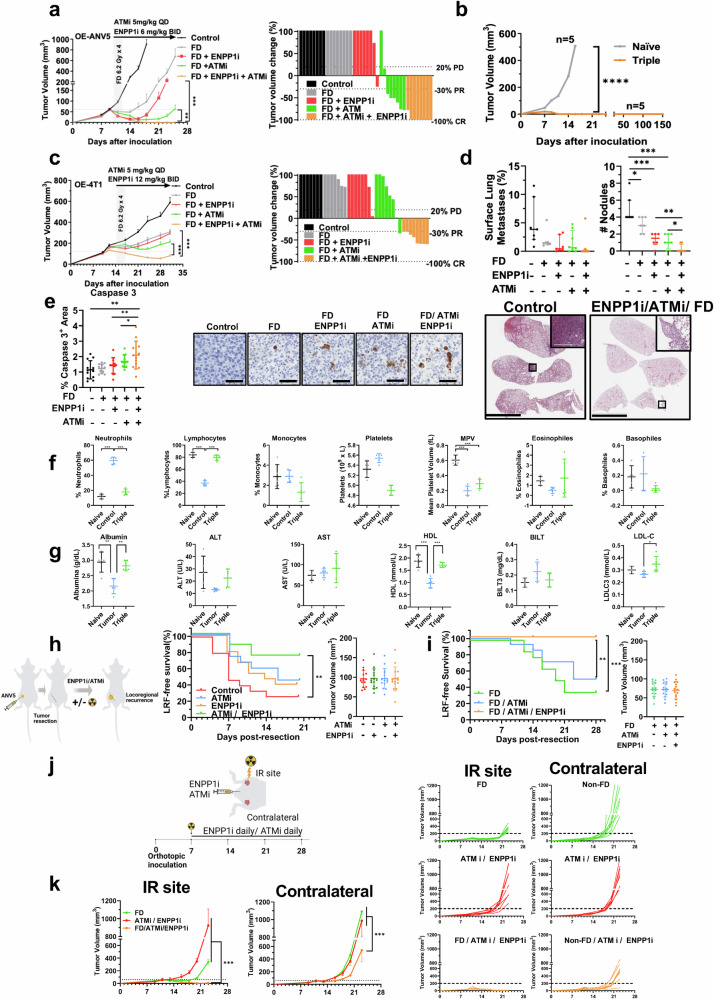
ENPP1i/DDRi post-IR eradicates local control and impacts disseminated disease. **a** *Left panel:* Tumor volume kinetics after orthotopic implantation of OE-ANV5 cells treated with FD (6.2 Gy × 4) alone or in combination with ENPP1i (6 mg/kg daily, BID), ATMi (5 mg/kg daily), or the triple combination (n = 8 mice/group). *Right panel:* Waterfall plot at the day of sacrifice. Kruskal-Wallis test was applied. Mean ± SEM are represented. **P < 0.01; *** P < 0.0001. **b** Tumor volume kinetics of orthotopic tumors in Control and dual-treated post-IR treated animals (n = 5 per group) which did not develop tumors were rechallenged by orthotopically implanting OE-ANV5 cells 2 months after treatment interruption. One-way ANOVA was performed. ****P < 0.00001. **c** *Left panel:* Similar experiment as in **a** using 4T1 cells. Treatments included ENPP1i (12 mg/Kg BID) and ATMi (5 mg/Kg daily). *Right panel:* Waterfall plot at the day of sacrifice. Kruskal-Wallis test was applied. Mean ± SEM are represented. ***P < 0.0001. **d** *Top panels:* Quantification of the metastatic surface (left) and the number of pulmonary nodules in histological section of mice treated performed by an in-house developed macro based on ImageJ®. Median and inter-quartile range are represented. Mann-Whitney U test was used for comparison. *P < 0.05; **P < 0.01; ***P < 0.0001. *Bottom panels:* Representative H/E images of lung lobules in Control and treated-mice with the triple combination. Scale bar = 5 mm. **e** *Left panel:* Quantification of Caspase-3 immunostaining in tumor sections of treated animals (n = 5/ group) for 4 days. *Right panels:* Representative images. Scale bar = 50 µm. **f** Quantification of hematological parameters in blood samples extracted from naïve animals compared to Control and triple-treated animals for 2 weeks of the indicated cell subpopulations. One-way ANOVA was used for comparison. MPV Mean Platelet Volume. **g** Quantification of plasma levels of the indicated biochemical markers. ALT Alanine aminotransferase, AST aspartate aminotransferase, HDL High density lipoprotein, BILT Bilirubin, LDL-C LDL-cholesterol. **h** *Left panel:* Schematic outline of LF assay. *Middle panel:* LF-free survival after tumor resection from OE-implanted cells. Mice (n = 15/group) were treated with ATMi (5 mg/Kg/day), ENPP1i (6 mg/Kg BID) or the combination from the day of tumor resection or treated with vehicle (control). *Right panel:* tumor volume at the day of tumor-resection (Day 0). Log-rank test was used in Kaplan-Meier curves. ** P < 0.001. **i** *Left panel:* LF-free survival after surgical resection of tumors derived from OE-ANV5 cells orthotopically implanted as in **h**. Mice (15 mice/group) were treated with FD (4 × 6.2 Gy) IR alone, on two consecutive days after surgery with an implanted catheter,^12^ in combination with ATMi (5 mg/kg daily) or with the dual ENPP1i (6 mg/Kg by oral gavage BID), and ATMi. *Right panel:* tumor volume at the day of surgery in each group. No differences in tumor margins between groups were detected. Log-rank test was used in Kaplan-Meier curves. **P < 0.01; ***P < 0.0001. **j** Experimental outline shows the orthotopic tumor cell inoculation in the irradiated mammary gland whereas the contralateral mammary gland was not irradiated. Animals were treated with ENPP1i and ATMi. **k** *Left panels:* Orthotopic tumor growth after double simultaneous inoculation of OE-ANV5 cells in opposite inguinal mammary glands in 3 groups of mice (8 mice/group). Treatments include IR only with fractionated dose (FD) performed in one flank, systemic ENPP1i/ATMi treatment, and triple treatment. Tumor volumes were monitored over time in both flanks. *Right panels:* Tumor volumes of each tumor at the IR flank and the non-IR contralateral flank. Kruskal-Wallis test was used for comparison. *** P < 0.0001

To further explore the impact of previous findings in vivo, we examined the tumor growth kinetics in several models of orthotopic tumor growth using OE cells to avoid the confounding effects elicited by other components of the transcriptomic signature present in CTC-in. Of note, ENPP1 tumor levels correlate with tumor aggressiveness.^12^ As expected, tumor growth delay was more pronounced in the FD group than in the Control group whereas FD in combination with ATMi induced a partial regression of several tumors. Furthermore, after treatment cessation, dual-treated animals showed a transient complete tumor regression in only 3 animals that eventually progressed (time to tumor detection >20 mm^3^) with a latency time of 16 days (Fig. 4a). Strikingly, in ATMi/ENPP1i post-IR-treated animals, we observed a complete tumor remission in 5 animals. After re-challenge performed 2 months after complete remission, these 5 animals showed complete and durable tumor rejection indicating long-lasting immune memory responses (Fig. 4b).

No major changes in vital organs were detected in triple-treated animals (Supplementary Fig. 4a). Similar results were replicated in OE-4T1 using a suboptimal ATMi (5 mg/Kg) dosage and a higher dose of ENPP1i (12 mg/Kg BID). Although FD radiation diminished tumor volume growth, double combinations FD/ENPP1i or FD/ATMi showed no additional effects in this cell line. Moreover, dual ATMi/ENPP1i post-IR achieved a stronger tumor regression than double FD/ATMi but did not eradicate any of the tumors at the indicated doses (Fig. 4c). Interestingly, analysis of spontaneous lung metastases also correlated with a reduction in the tumor surface and number of nodules in the triple treatment as compared to controls, indicating a marked effect impacting distant dissemination (Fig. 4d). Of note, in an independent experiment using a higher dose of ATMi (15 mg/Kg daily) and identical dose of ENPP1i (12 mg/kg BID), a remarkable reduction in tumor volume was observed after the triple treatment, amounting to an almost complete eradication of tumors during treatment indicating a dose-dependent effect (Supplementary Fig. 4b, c). As expected, a marked tumor apoptosis was revealed by caspase-3 immunostaining in post-IR ENPP1i/ATMi-treated tumors for 5 days derived from OE-ANV5-inoculated cells (Fig. 4e). Blood cell counts revealed comparable numbers in triple-treated mice to those of naive mice. Both treated and control tumor-bearing mice displayed a reduced mean platelet volume when compared to naïve mice. No significant differences were found in other cell subpopulations across the groups (Fig. 4f). Biochemical markers in plasma showed no major changes between naive and triple-treated animals (Fig. 4g).

Next, we examined whether the antitumor effect elicited by ENPP1i/ATMi alone or post-IR could have an impact eradicating residual and/or CTC engrafting in the RTB. We selected a suboptimal dose of ATMi (<5 mg/Kg) to avoid any toxicities and to detect potential additive effects with ENPP1i.

Initially, we tested dual ENPP1i/ATMi in previously developed LF models^12^ (Fig. 4h). Interestingly, we observed a significant rise in LF-free survival rates in the dual treatment as compared to single treatments (Fig. 4h), indicating an additive effect in this LF model. Addition of IR to dual blockade could further improve LF over the current locoregional standard of care (surgical resection followed by fractionated-dose, FD). Remarkably, addition of IR in conjunction with this double inhibition completely eradicated LF as compared with dual combinations, indicating a profound effect completely obliterating local recurrence (Fig. 4i). Of note, no differences in resection margins or tumor volume were detected in excised tumors (Fig. 4h, i and Supplementary Table 1).

Next, we examined the induction of potential abscopal effects in an experimental setting outlined in Fig. 4j. Interestingly, 21 days post-IR, ENPP1i/ATMi induced a dramatic reduction in contralateral tumors as compared to only IR-treated or dual ENPP1i/ATMi treated, indicating enhanced antitumor immune responses and a marked abscopal effect (Fig. 4k).

Thus, concurrent ATM/ENPP1 blockade boosted radiation effects leading to better local tumor control. This treatment also elicited abscopal effects and diminished the burden of metastases, presumably by enhancing antitumor immunity impacting disseminated disease.

### ENPP1 and ATM blockade post-IR elicits STING activation and adaptative antitumor immunity

We postulated that STING activation mediated by ENPP1 blockade could account for the induced antitumor immune responses previously observed.^21^

**Fig. 5:**
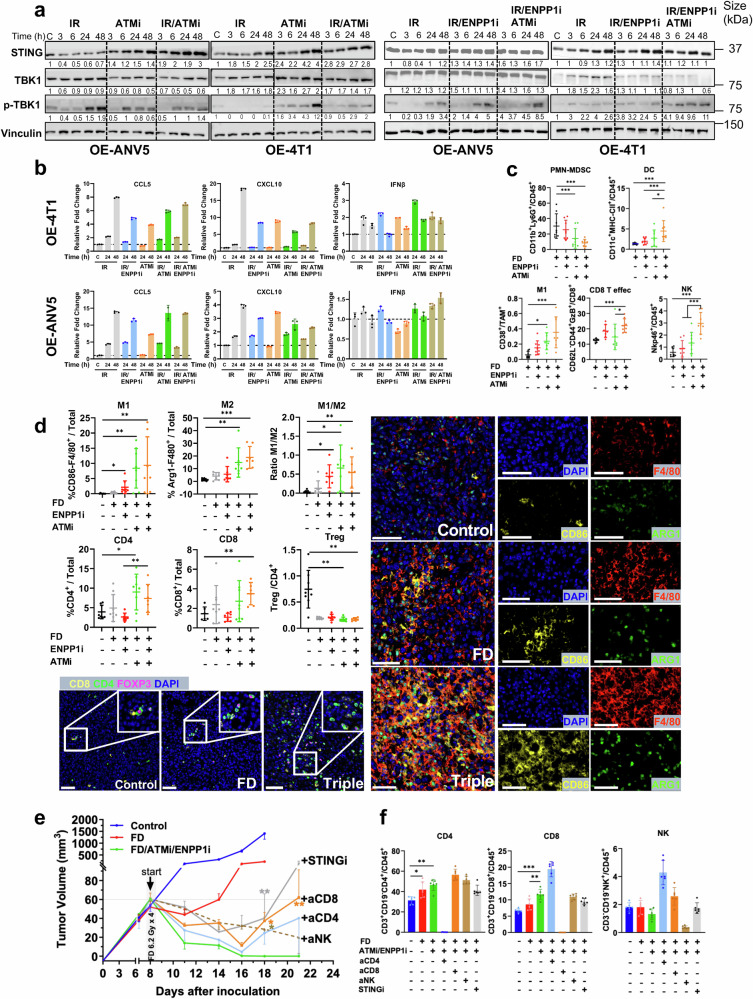
ENPP1i / DDRi post-IR boosts anti-tumor immune responses. **a** Immunoblot analysis of protein expression levels of STING, TBK1, phospho-TBK1 and Vinculin from cells lysates of OE-ANV5 and OE-4T1 cells treated with IR (2 Gy), ENPP1i (5 µM) and ATMi (5 µM) extracted for the indicated time post-IR. Data are representative of three independent experiments. **b** Expression levels assessed by RT-qPCR of the indicated genes in OE-4T1 and OE-ANV5 cells treated as indicated. **c** Quantification by flow cytometry of tumor-infiltrating immune subpopulations derived from orthotopic tumors at day 4 post-IR from tumor cell inoculation treated with ENPP1 and ATMi daily, showing the reverted immunosuppression. Mean and SD are represented. Kruskal-Wallis test was used for comparison. **d** Quantification of the multispectral analysis performed for the indicated subpopulations of myeloid M1 and M2 macrophages (top) and (bottom) lymphoid CD4^+^, CD8^+^ T cells and CD4 regulatory T cells. Mean and SD are represented. Representative images from multispectral fluorescence analysis in the indicated groups, showing the spatial resolution of different immune myeloid subpopulations (*right*), and lymphoid (*bottom*) infiltrating the tumor core in the group treated with FD or FD plus ENPP1i/ATMi (Triple). Scale bar = 50 µm. Kruskal-Wallis test was used in all panels, except for CD8^+^T cells where Student´s t-test was used for comparison. **e** *Left panel:* Tumor volume kinetics after orthotopic inoculation of OE-ANV5 tumor cells. Mice (n = 8 mice/group) were treated when tumors reached a 70 mm^3^ volume, with vehicle (Control), FD (6.2 Gy × 4) and triple combination of FD/ ENPP1i (6 mg/Kg BID)/ ATMi (5 mg/Kg). In other groups of mice, in addition to the triple combination, mice were treated with anti-CD8, anti-NK1.1, anti-CD4 depleting antibodies (200 µg of antibodies, three times per week) and STING inhibitor (i) (C-176, 5 mg/Kg i.p. daily). Kruskal-Wallis test was used for comparison of depleting antibodies against triple treatment. **f** Quantification of the indicated immune subpopulations in the blood for each group of mice at the day of sacrifice. *P < 0.05; **P < 0.01; ***P < 0.0001

To explore this postulate, we examined the phosphorylation levels of cGAS-STING signaling effector TANK-binding kinase1 (p-TBK1). The induction of STING by ATMi was not further enhanced by its combination with IR in both cell lines (Fig. 5a). Interestingly, ENPP1i incubation in combination with IR showed an increased cGAS-STING signaling effector TANK-binding kinase1 (TBK1) at 48 h. In addition, incubation with ATMi/ENPP1i post-IR led to a dramatic increase in phospho-TBK1 levels as early as 3 h and was sustained until 48 h in both cell lines (Fig. 5a). Furthermore, expression levels of downstream effectors of STING activation were upregulated, particularly CCL5 and CXCL10, upon ATMi post-IR or triple treatment (Fig. 5b, Supplementary Fig. 4d). Collectively, this data indicates that ATMi added to dual ENPP1i/ IR triggers a prompt and robust STING signaling activation in tumor cells.

To further evaluate whether STING activation could also have an impact in immune subpopulations in vivo, we dissected the immune landscape and evaluated the antitumor responses in independent experiments. Immunophenotyping of tumors obtained in orthotopically inoculated animals treated with ATMi/ENPP1i 4 days post-IR showed a marked decrease in polymorphonuclear myeloid-derived suppressor cells (PMN-MDSC) and a concomitant increase in M1-polarized macrophages, natural killer (NK) cells with a significant increase in CD8^+^ T effector and dendritic cells (DC), indicating an increased antitumor immunity (Fig. 5c), compared to the immune exclusion observed in control tumors. Notably, the immune exclusion seen in tumors derived from OE-ANV5 was consistent with the expression of several immunosuppressive cytokines detected in OE-ANV5 versus ANV5 cells in vitro (Supplementary Fig. 4e).

Concordantly, multispectral fluorescent analysis using a different set of antibodies revealed a marked prevalent increase in M1 over M2-polarized macrophages in triple treatment. Importantly, this was accompanied by a significant infiltration of CD4^+^ T and CD8^+^ T cells in the tumor bulk of double-treated post-IR animals (Fig. 5d) (Supplementary Fig. 5a, b).

Interestingly, depletion of NK, CD4^+^, CD8^+^ T cells, and STING inhibition did not show significant effects on tumor growth kinetics as compared to triple-treated animals for 8 days (up to day 16). After this point, from day 16 to day 18, depletion of CD4^+^ and CD8^+^ T cells in triple-treated mice showed 3- and 5-fold increased tumor volumes, respectively, whereas a significantly marked 1.2-fold increase was observed in STINGi-treated mice (p < 0.01). In contrast, NK depletion did not significantly influence tumor volume growth between day 16 and 18, although tumor volumes were significantly enhanced as compared to triple treatment (p < 0.05). Furthermore, STING pathway inhibition (C-176) in ATMi/ENPP1i treated tumors post-IR led to a 4-fold increase in tumor volume at day 21 as compared to day 16, whereas a 5- and 9-fold increase was observed in CD8^+^T and CD4^+^ T depleted groups, respectively (Fig. 5e). At the final day of the experimental period, the group of CD8^+^T depletion showed a significant increase in tumor volume as compared to triple treatment (p < 0.01), indicating the marked involvement of CD8^+^T cells and a minimal role of NK cells mediating the antitumor immune responses. A substantial STING-mediated effect and a partial effect mediated by CD4^+^ T cells were detected in the antitumor immunity. Interestingly, triple treatment led to a 6/6 cured mice, whereas, 4/6 and 2/6 were found to be cured in STINGi and CD4^+^T depleted groups, respectively. In contrast, only one animal was cured in the group of NK depletion and none in the CD8^+^T depleted group. Despite the impact on tumor volumes in groups treated with STING inhibition and CD4^+^ T cell-depletion, these groups did not reach statistical significance at the end of the experimental period as compared to the triple treatment group, an event most likely related to the high number of cured mice observed in these groups, which limits the statistical significance.

Notably, specific depletion of the target subpopulations was efficiently achieved, as shown by flow cytometry analysis of blood samples (Fig. 5f). These findings suggest that this triad elicits strong antitumor immune-mediated effects markedly involving CD8^+^ T cells and the activation of the STING pathway, and partially dependent on CD4^+^T cells.

Taken together, these results substantiate the relevance of non-cell autonomous effects involving immune cell subpopulations in the antitumor effect triggered by the ATM/ENPP1 blockade post-IR.

### Identification of ENPP1^+^ gene signature linked to radioresistance in human breast cancer

To examine the translational significance of the identified ENPP1^+^ gene signature associated with radioresistance, we explored to which extent the murine gene subset identified in murine CTC-in could be sustained in human tumors during tumor-stromal co-evolution. To this end, we investigated human ER^+^, HER2^+^ and TNBC specimens by reanalyzing single cell-RNA sequencing (scRNA-seq) data.^22^

**Fig. 6:**
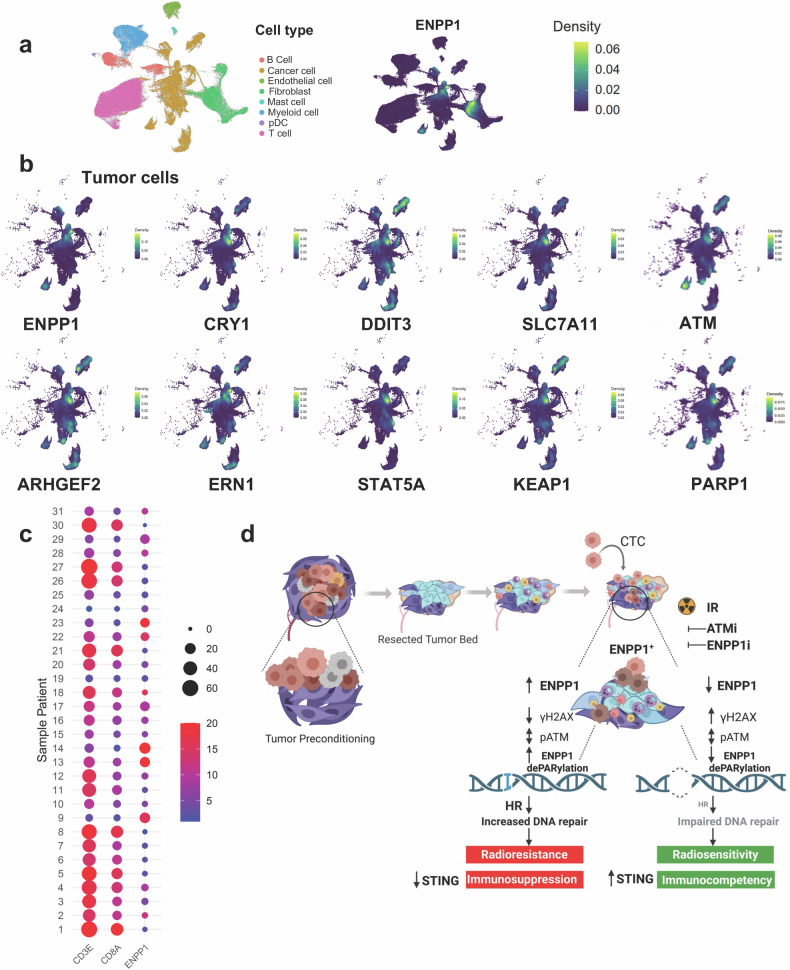
Identified ENPP1^+^ gene signature is sustained in human breast cancer tumors. **a** Uniform Manifold Approximation and Projection (UMAP) of scRNA-seq data of 31 human breast cancer tumors showing the different cell compartments (*Left panel*) and the ENPP1 expression levels (*Right panel*). **b** UMAP of the tumor cell compartment showing the upregulated expression of the indicated genes that overlap with expression of ENPP1 in tumor cells. **c** Dot plot for the gene features (CD3E, CD8A and ENPP1) in each sample patient from EGAD00001006608 scRNA-seq dataset.^22^ Point size reflects the percentage of gene expression (pct.exp) for the corresponding feature in each sample. The color scale indicates the average scaled gene expression (avg.exp.scaled) of each feature. **d** Schematic representation of the acquisition of ENPP1^+^ phenotype leading to radioresistance. *Top left*: During primary tumor growth, tumor cells precondition local and distant sites. After resection, remaining preconditioned cells along with wound repair events, create a host niche for the engraftment of residual cells and/or CTC that acquire a gene transcriptomic signature characterized by enhanced genome integrity and stem-like features. In this preconditioned environment, engrafted cells with the acquired ENPP1^+^-transcriptomic signature exhibit a resistant phenotype mediated by changes in dePARylation and phopho-ATM kinetics, thereby favoring HR-mediated DNA damage repair. This mechanism endows cells ´ability to overcome IR-mediated genotoxic stress. Concurrent inhibition of ENPP1 and ATM post-IR impairs DNA repair and boosts immunocompetency by eliciting STING activation in both tumor and non-tumor cells demonstrating a therapeutic susceptibility (Figure generated by BioRender)

Single-cell analysis identified subpopulations of tumor cells expressing high ENPP1 levels in all tumor subtypes and in a fraction of carcinoma-associated fibroblasts (CAF), suggesting the involvement of ENPP1 activity in the tumor milieu (Fig. 6a). Other ENPP1^+^-cells include the myeloid and T cell compartments (Supplementary Fig. 6a).

We visualized using Uniform Manifold Approximation and Projection (UMAP) whether components of the upregulated gene signature previously identified superimpose with ENPP1 in TNBC tumors. Interestingly, a cluster of ENPP1^+^ tumor cells overlaid DDIT3, CRY1 and SLC7A11, whereas a small fraction of ENPP1^+^ tumor cells tangentially superimposed with ERN1, STAT5A, ARHGEF2 and KEAP1 gene expression (Fig. 6b). Remarkably, a subset of ENPP1^+^ tumor cells showed elevated levels of ATM and PARP1, suggesting an enhanced resilience to stress (Fig. 6b). Other genes marginally overlaying with ENPP1 include TIMELESS, SEMA4F, ALPL and VEGFC (Supplementary Fig. 6b). Remarkably, we observed an inverse correlation between tumor ENPP1 expression levels and the presence of CD3ε and CD8A immune T cells, in line with previous findings (Fig. 6c). Of note, we found strong correlation between the gene expression levels of ENPP1 and CRY1 (R = 0.73, p = 3.6 × 10^−6^) and DDIT3 (R = 0.65, p = 7.4 × 10^−5^) in patients (Supplementary. Fig. 6c). Overall, these findings indicate a partially conserved cross-species signature of ENPP1^+^ tumor cells that confers an immunosuppressive tumor microenvironment.

Additionally, the Louvain algorithm identified 8 clusters that were visualized using UMAP, one of which (myCAF) contains almost the majority of ENPP1^+^ expressing CAF (Supplementary Fig. 6d). The myCAF subset has been shown to be localized near the invasive tumor edge and to co-express a gene myofibroblast cluster including COL1A2, TAGLN, LRRC15, and GJB2 genes. ENPP1^+^ CAF also co-express FAP and ITGB suggesting a functional cross-talk in the tumor microenvironment.

Collectively, these findings indicate that ENPP1^+^ gene signature expressed in human TNBC by a fraction of tumor cells may confer heightened resilience to genotoxic stress and promote an immune landscape that favors treatment resistance.

## Discussion

In this study, we identified a conserved ENPP1^+^-phenotype associated with enhanced resilience to genotoxic stress.^23^ This phenotype correlates with a multigene signature co-expressed with ENPP1 (Fig. 6d) which is also conserved in human breast cancer, underscoring its functional co-implication and significance.^24^ This approach allowed us to identify ENPP1-mediated intrinsic tumor functions, including tumor cell radioresistance, thereby highlighting a direct link between ENPP1 and genome integrity.

ENPP1 depletion post-IR resulted in high cGAMP levels, whereas cGAS silencing impairs cGAMP synthesis, indicating that cGAMP levels are not directly involved in radioresistance, despite the fact that cGAS silencing mirrored the effects of ENPP1 depletion on IR-sensitivity. Previous findings indicate that depletion of cGAS impairs recognition of cytosolic DNA, increasing replication stress and leading to increased radiosensitivity.^25^ In contrast, nuclear cGAS translocation also compromises DNA repair by interacting with γH2AX and disrupting the PARP1-Timeless complex at DSB, ultimately leading to reduced HR.^21^ Thus, the impaired functional damage repair could be partially explained by changes affecting the function of ENPP1-elicited dePARylation, a post-translational modification modulating the hydrolysis of adenosine diphosphate ribose (ADPr), altering the recruitment and the kinetics of DDR components including ATM and PARP1.^26,27^ ENPP1-depletion could tilt the PARylation homeostasis of several proteins of DDR resulting in an unanticipated vulnerability to genotoxic stress. In fact, nuclear phospho-ATM kinetics were perturbed in OE cells subjected to oxidative stress conditions in a cell-specific manner which were reverted by ENPP1i. Remarkably, PARG, another functionally related dePARylating enzyme, has emerged as a target modulating DNA repair^28,29^ and its inhibition sensitizes tumor cells to IR promoting cancer cell death.^30^

In addition, STING-activation induced by ENPP1 depletion elevates reactive oxygen species (ROS) levels indirectly regulating the susceptibility for subsequent DNA damage,^31^ effects that are further exacerbated by IR.^32^ This highlights the role of ENPP1 inhibition in increasing susceptibility to DNA damage.

The mechanisms by which residual cells or engrafted CTC acquire and sustain an ENPP1^+^-gene signature remain to be clarified. Intriguingly, ENPP1 levels were found to be elevated following in vitro exposure to IR, an event that may be part of the oxidative stress response induced by IR and that triggers inflammatory pathways. This acute inflammatory stress response could result in compensatory transcriptional increase in ENPP1 to hydrolyze excess ATP and GTP levels generated both intra- and extracellularly. This suggests that surviving cells post-IR could be endowed with a more resistant phenotype. Additionally, at local or distant sites, a tumor-primed environment created during tumor growth, along with infiltrated immune-stromal components, could provide a supportive milieu for tumor cell engagement. In this context, responses to the imposed metabolic, hypoxic, and microenvironmental stress conditions during tumor cell engraftment may enhance tumor cell resistance through the induction of pro-survival and transition from suspension-to-adherent states^33^ by incorporating stem-like and resilient traits.^34^

Given its prominent role in radioresistance, ENPP1 blockade unveils a novel vulnerability by affecting core DNA damage repair functions, specifically enhancing radiosensitivity through impaired HR, especially when DSB, further exacerbated by IR, rely on a compensatory error-prone NHEJ pathway. The acquired HR-deficient phenotype elicited by ENPP1i enhances DDRi sensitivity in DDRi-resistant cells (BRCA-competent cells). Thus, our study supports the notion that ENPP1i emerges as a sensitizer to DNA-damaging agents. This underscores the possibility of incorporating ENPP1i in a fraction of TNBC patients, for whom DDRi is standard of care, which may prove clinically advantageous. A consequence of the cytotoxic effects observed in combined ENPP1i/ATMi is the potential to mimic the HR-deficient phenotype seen in genetic defects of HR signaling components^35^ or other mutations, such as BRCA mutations,^36^ which could sensitize tumor cells to this treatment. Therefore, a more salient outcome could be anticipated in specific subsets of patients. Furthermore, additive effects have also been observed when combining DDRi with targeted inhibitors of other actionable oncoproteins in the MEK and PI3K signaling pathways, all of which involve HR repair.^37^ This suggests that ENPP1 blockade could have broad implications in combined anti-cancer therapies. Indeed, synergistic effects have been reported with Olaparib, Paclitaxel, and anti-PD-L1.^38^

Beyond the tumor cell-autonomous effects impairing HR upon ENPP1 blockade, STING activation triggers a non-cell autonomous induction of an adaptative immune response that impacts both local and distant dissemination. Several factors may cooperatively contribute to the robust STING activation observed. First, DDR blockade elicits cGAS-STING engagement through the spurious accumulation of resected DNA fragments, further exacerbated by IR, leading to the emergence of micronuclei in the cytosol^39,40^ and the release of mitochondrial DNA.^41^ Second, STING signaling is also activated by the ENPP1i-mediated cGAMP accumulation. The relevance of STING was underscored in vivo, as STING depletion reestablished tumor growth in the obliterated triple-treated tumors. In light of this accumulated evidence, ENPP1 links DNA genome integrity to tumor immune tolerance through its role in the STING pathway.

These findings could significantly impact the oligometastatic setting, particularly when paired with subsequent IR treatment at distant sites within the context of dual ENPP1i/DDRi. The potential to affect incipient metastases that express ENPP1 is also supported by the long-term immunologic memory observed. Furthermore, STING activation not only hinders the reactivation of dormant metastases, thereby preventing disease relapse, but could also eliminate disseminated tumor cells.^42^ Consequently, our findings reinforce the notion that optimal management for selected patients with disseminated disease may be best achieved through a combination of local and systemic therapies, by the application of IR at both local and distant sites to elicit antitumor immune responses in the context of dual inhibition.^43^

Our study constitutes a valuable proof-of-principle opening other potential opportunities for its translation into the clinical setting. Since other sources of IR elicit more deleterious effects (High frequency of closer DSB) upon ATMi treatment by impeding the repair of dense DSB,^44^ greater anti-tumor effects with less toxicities could potentially be expected in ENPP1i/ATMi treatment with these sources as compared to the X-Rays used in our models, but the immune response under these modalities remains to be addressed. Furthermore, one could anticipate that immune checkpoint blockade (ICB) could sequentially be paired with IR/ENPP1i/DDRi to boost anti-tumor adaptive immunity or could be used to build more robust antitumor immune responses.^45^ In this regard, patients with low ENPP1 expression are better responders to ICB.^46^

Even in tumors with ENPP1-devoid cancer cells, dual combination post-IR could yield antitumor benefits by blocking functional ENPP1 in CAF and immune cells, thus reversing immunosuppression, and by eliciting cGAS-STING signaling in a subset of ENPP1^+^-non-tumor cells. It could also cooperatively affect phagocytes where the DNA internalization from dying IR cells enhances cGAS, among other mechanisms.^47^ In addition, DNA repair inhibitors act as “radiosensitizers” that, in combination with IR, promote more salient effects than single treatments. The synergy with the cell-permeable ENPP1i offers the additional benefit of a lower required dosage of ATM or PARP1 inhibitors, preventing their inherent toxicities with uncompromised antitumor efficacy (Supplementary Fig. 3e). In support of this, ATMi was used at suboptimal doses in vivo to avoid any toxic effects and to uncover additive mechanisms. Moreover, in vivo analysis of lymphopenia, an event associated with post-IR toxicity in wide-field treated patients, was not observed in our models. Notably, non-cancerous breast cells were also unaffected in vitro by the combination.

Another potential advantage of STING modulation by the bi-daily ENPP1i administration (half-life ∼6 h, unpublished observation), is that it could lead to a metronomic induction of STING and IFN-responses, preventing the detrimental hyperactivation effects sporadically observed by sustained STING agonist signaling.^48^ Paradoxically, persistent STING signaling after chronic cGAMP stimulation was skewed towards immunosuppressive ER stress signaling and non-canonical NF-κB cascades rather than interferon responses, resulting in tumor progression and metastasis.^49^

In summary, our study uncovers the unanticipated involvement of ENPP1 within the proficient DNA integrity traits acquired by resilient cells to overcome genotoxic treatment constraints. This finding was leveraged to identify novel combined susceptibilities to enhance radiosensitivity and innate and adaptive immune responses, as a powerful proof-of-principle to abrogate tumor growth, eradicate LF, and impact distant dissemination. This study will enable new mechanism-based combination trials, opening innovative avenues in the clinical management of local recurrence in TNBC and other solid tumors.

## Material and methods

All in vivo experiments were performed in compliance with institutional guidelines approved by the Local Animal Ethics Committee (CEEA114-19) according to European Council Guidelines.

### Cell lines

4T1 cells were obtained from ATCC and Antigen-negative variant 5 (ANV5) cells were originally derived from relapsed tumors after subcutaneous inoculation of mouse mammary carcinoma (MMC) cells into non-transgenic FVB/N mice (a kind gift of K.L. Knutson). 4T1 cells were cultured in RPMI 1640 medium, as ANV5 which was additionally supplemented with 10 mM HEPES, 1% GlutaMAX®, and 1 mM sodium pyruvate. Authentication was performed periodically by amplification of STRs compared to known standards. The cells were tested monthly for *Mycoplasma* infection using MycoAlert®. Gene silencing was performed by lentiviral infection using shRNA in pLKO.1 vector (Addgene 52920) as previously described.^50^ For gene overexpression, mouse cDNA encoding Enpp1 was cloned into pBABE-Neo as described (Addgene 1767).

### Reagents and drug treatments

Antibodies used are included in Supplementary Tables 1–4. A permeable ENPP1i (AVA-NP-695) was generously provided by Avammune Therapeutics Inc. ATMi (AZD1390) and the other inhibitors were purchased at MedChem Express. Both ENPP1i and ATMi were dissolved in 0.5% methylcellulose 400 (Sigma) in tap water and administered by oral gavage.

### Clonogenic assay

Cells in suspension at 312 to 10 000 cells per well were IR at different doses and seeded in triplicate in 6-well plates with the indicated drug treatments. After 7 days cells were washed with cold PBS twice, fixed with 4% PFA (Panreac) for 15 min at RT, and colonies were stained with Crystal Violet solution (Sigma-Aldrich) for 15 min, plates were scanned, and colonies were counted manually using and imaging Fiji software (ImageJ®). Plating efficiency (PE) was calculated after seeding 156, 312, and 625 cells in 6-well plates for 7 days using the following formula: PE = (Average “n” colonies per well)/(N cells seeded). Survival fraction at X Gy (SFX) was calculated as follows SFX= (Average “n” colonies per well at X Gy)/(N cells seeded 0 Gy*PE). Survival curves were generated by fitting the surviving fraction to a linear-quadratic model: SF = exp( −αD −βD2), where SF is the surviving fraction and D is the dose. D_50_ values were calculated by solving the resulting equations for survivals of 50%.

### Cell survival assay

Cells in suspension at 5000 cells/mL density were IR at the indicated doses (0–12 Gy), seeded at 250–500 cells per well, depending on the cell line in 96-well plates, and incubated with the indicated drugs for 5 days. On day 5, cells were fixed and stained with Crystal violet as previously described. Stained cells were dissolved in 20% acetic acid, and absorbance was measured at 570 nm using a SpectroStar Nano (BMG Labtech, Germany). Results were normalized against the non-treated (Medium/DMSO control) wells.

### Target pharmacological screen using DNA damage repair inhibitors (DDRi)

Drug screening was performed in a matrix (n = 3/condition) consisting of ENPP1i (IC_20_ = 2.72 µM and IC_30_ = 3.62 µM) in ANV5-OE, or selected DDRi or the respective combinations. OE-ENPP1 ANV5 cells were seeded at 500 cells per well in triplicate in 96-well plates. Drugs were added 24 h post-seeding. On day 5, cells were fixed with 4% PFA and stained with 1% crystal violet. Stained cells were dissolved in 20% acetic acid and absorbance was measured at 570 nm. The list of DDRi can be found in Supplementary Table 5.

### Statistical analysis

GraphPad Prism v.10.2.3 was used for statistical analysis. Data were tested for normality, and for experiments with two conditions, a two-tailed unpaired t-test with Welch’s correction was used to determine the P value, while for experiments with more than two conditions, a one-way ANOVA test with Dunnett’s multiple comparison test was used. When data were not normally distributed, a Mann–Whitney test (two samples) and a Kruskal–Wallis with Dunn’s post hoc test (multiple comparison) were used instead. A P value of ≤0.05 was considered significant. Surviving fraction curves adjusted to the quadratic model were compared using extra sum-of-squares F test. All graphs show at least three biological replicates (independent experiments) unless otherwise stated.

## Supplementary information

Supplemental Material

Data set containing uncropped Gels

## Data Availability

RNA-seq data have been deposited under the accession code GSE277249.
