# Supplementary material for: Dual ENPP1/ATM depletion blunts DNA damage repair boosting radioimmune efficacy to abrogate triple-negative breast cancer
Source: Signal Transduct Target Ther. 2025 Jun 13;10:185. doi: 10.1038/s41392-025-02271-2 (PMC12162836; doi:10.1038/s41392-025-02271-2)
Supplement: Supplementary file 1 — Supplemental Material [file 41392_2025_2271_MOESM1_ESM.docx]

Supplementary Materials for

**Dual ENPP1/ATM depletion blunts DNA damage repair boosting**

**radioimmune efficacy to abrogate triple-negative breast cancer**

Borja Ruiz-Fernández de Córdoba§, Karmele Valencia§, Connor Welch, Haritz Moreno, Susana Martínez-Canarias, Carolina Zandueta, Eduardo Gómez, Alfonso Calvo, Nerea Otegui, Mirari Echepare, Ignacio Garzón, Daniel Ajona, David Lara-Astiaso, Elisabet Guruceaga, Laura Guembe, Rubén Pío, Ignacio Melero, Silvestre Vicent, Fernando Pastor, Rafael Martínez-Monge*,

Fernando Lecanda*

Correspondence to: [rmartinezm@unav.es](mailto:rmartinezm@unav.es) and [flecanda@unav.es](mailto:flecanda@unav.es)

**This PDF file includes:**

Materials and Methods

Figures. S1 to S7

Tables S1 to S8

MATERIAL AND METHODS

**Orthotopic injection**

All experiments were performed in compliance with institutional guidelines approved by the Local Animal Ethics Committee (CEEA114-19) according to European Council Guidelines. All experiments were executed in female mice since sex did not represent a biological variable in this study. Mice were anesthetized by using isoflurane. Local shaving and disinfection were performed in the area surrounding the 4^th^ inguinal mammary gland. A small longitudinal incision of 0.5–1 cm was performed 2 mm adjacent to the nipple. A total of 50 μl (25 μl Matrigel and 25 μl of Cell suspension in PBS) containing 2 x 10^4^ ANV5 or 4T1 cells were then slowly delivered into the mammary gland of 6- to 7-week-old syngeneic FVB/N or Balb/C mice (Envigo, Barcelona, Spain), respectively, using a 0.3 ml insulin syringe with 29 G needle. The skin was sutured with absorbable surgical 6–0 silk (Vicryl). To evaluate orthotopic tumor growth kinetics, tumor size was measured manually with a digital caliper and tumor volume was calculated using the ellipsoid formula *V* = π/6 (*L* × *W* × *H*). Maximal tumor volume permitted was <1500 mm^3^. To evaluate local tumor recurrence, when tumors reached the indicated volume, tumors were resected *en bloc* and immediately ink-marked and fixed for pathological evaluation. Surgical margin assessment is summarized in **Table S1**. Lung metastases were analyzed by H/E histological evaluation and quantification of metastatic area and total lung area and the number of metastatic nodules, by using ImageJ analysis.

**Isolation of Circulating Tumor Cells (CTC)**

Briefly, 4T1 cells were lentivirally transduced to express GFP for tracking and isolation. After intramammary inoculation in immunodeficient mice (Rag2tm1.1Flv Il2rgtm1.1Flv/J), tumors were resected at the appropriate time. Total blood was extracted under anesthesia, prior to sacrifice, and mixed with complete medium in p-150 plates. Tumor cells (CTC evading the tumor or CTC-out) were selected with puromycin. For ANV5, CTC-out could not be isolated because of the low numbers of CTC present in this model. To obtain CTC engrafting in the resected tumor bed or CTC-in, ANV5 parental cells or 4T1 CTC-out were inoculated in the left cardiac ventricle in 6-week-old female immune-compromised mice. Tumors were disaggregated to single-cell suspension, and after selection with 4-6 µg/mL of puromycin, CTC-in were cultured until single-cell derived colonies were detected.

**In vivo depletion of immune subpopulations**

In vivo depletion of CD8^+^, CD4^+^ T cells and natural killer (NK) cells was achieved by weekly intraperitoneal injections (100 μg per mouse, 3 times per week) of anti-mouse CD8α (clone YTS 169.4), anti-mouse CD4 (clone GK1.5), and anti-mouse NK1.1 (clone PK136) antibodies, respectively (Bio X Cell). Rat IgG2a isotype (clone 2A3) antibodies were used as control (Bio X Cell). Survival was monitored daily, and tumor volume was measured until maximum allowed size or at the end of the experimental period.

**Fluorescent Traffic Light reporter (TLR) system**

This system described elsewhere ^1^ includes a viral transduction system composed by I-SceI-T2A-IFP (endonuclease restriction enzyme), the Donor-T2A-BFP (Donor template) and the TLR (Reporter system). Briefly, TLR generates a readout for homology-directed repair (HR) mediated gene targeting and mutagenic non-homologous end joining (mutNHEJ) mediated gene disruption occurring at a designed individual DNA breakpoint. This fluorescent reporter is designed as such, as a double strand break (DSB) is produced at an embedded I-SceI nuclease cleavage site. The DSB repair generates distinct fluorescent signals upon resolution either through HR (where a functional green fluorescent protein eGFP open reading frame is reestablished) with the aid of the exogenous donor template or through mutNHEJ (where non-repaired GFP DSB, places a mCherry coding sequence in frame to signal gene disruption). We transduced cells containing a single copy of the TLR integrated into the genome, transduced with I-SceI with or without the donor template transduction. After 72 h, cells were analyzed by flow cytometry. Cells acquiring the Donor template were sorted by V450-PB-A, whereas cells transduced with I-SceI were positive for R712-APC 700A. Cells transduced with TLR positive for I-SceI and Donor template were analyzed for Cherry (Y610-Cherry) and FITC (B525-FITC). The percentage of cells with FITC and Cherry were referenced to total cells (positive for V450-PB-A and R712-APC 700A).

**Comet Assay**

DNA damage was evaluated using the CometAssay® (#4250-050-K) (Biotechne, R&Dsystems) using the alkaline electrophoresis method. Cells were treated with IR, ENPP1i, or the combination for 24 h. Treated cells (500-1000) were seeded CometSlide™ according to the manufacturer’s recommendations. Quantitative data were obtained using an automated macro developed in Fiji software (ImageJ®).

**Immunofluorescence staining**

Cells were seeded in 6-well plates containing coverslips. Following IR and adequate incubation time to allow for DDR, cells were fixed with 4% formaldehyde in PBS for 15 min, and permeabilized with 1%Triton X-100 PBS solution for 30 min. After blocking with 2% BSA for one hour, cells were washed for three times in PBS and subsequently incubated with anti-γH2AX antibody (Cell Signaling, 9718; 1:200) overnight. Next day, cells were incubated with anti-rabbit IgG-Alexa Fluor 555-conjugated antibodies (Invitrogen, A31572; 1:200) for 30 min to 1 h at room temperature and cells were stained with 100 ng/mL 4, 6-diamidino-2-phenylindole (DAPI, Biotium, 40009; 2,5 µg/ml) for one hour to visualize DNA. The cover slips were rinsed, mounted onto glass slides using antifade solution and sealed. Imaging was performed with a Zeiss Axiovert 200 microscope (Zeiss) equipped with a Perkin Elmer Spinning disk unit, a Hammamatsu C9100-50 CCD camera, and a 40× Plan Apochromat Objective (N.A. 0.95). Quantitative data were obtained using an automated macro developed in Fiji software (ImageJ®).

**Preparation of Protein Lysates for PARylation Assay**

Cell culture conditions were consistent with previously described methods. On day -1, 1,000,000 cells were plated in a 100 mm culture dish. Starting 24 hours later (day 0), the existing media was discarded, and cells were exposed to freshly prepared media containing the treatment agent (5 mM H2O2). Cells were incubated with H_2_O_2_ for periods ranging from 10 min to 2 h. At the end of the treatment, cells and supernatants were separated and centrifuged at 1,200 rpm for 5 minutes. The supernatant was then removed, and the cell pellets were lysed with lysis buffer as previously outlined^2^, using RIPA buffer [1% Nonidet P-40, 50 mmol/L Tris-HCl (pH 7.4), and 150 mmol/L NaCl] enriched with protease inhibitors (Sigma) and 10 μM PJ34 (Thermo Fisher Scientific) to prevent PARP1 activation during lysate preparation

**Radiation procedures**

Cell DNA damage was induced by ionizing radiation (IR) with the indicated Gray (Gy) using a Nordion GammaCell 3000 irradiator. For non-invasive application of orthotopic tumors, animals were irradiated with a dual-focus-X-Ray tube external beam on a small animal radiation research platform (SARRP) from XStrahl, at 220kV and 13 mA, at the indicated doses (**Sup. Fig. 7**). In fractionated irradiation, 4 fractions of 6.2 Gy each, twice a day, at least 6 h apart, were delivered over two consecutive days. Single dose irradiation of 15 Gy and fractionated irradiation of 24.8 Gy (6.2 Gy x 4) are equi-effective as per the Linear-Quadratic formulation.

**REFERENCES**

1 Certo, M. T. *et al.* Tracking genome engineering outcome at individual DNA breakpoints. *Nature methods* **8**, 671-676 (2011).

2 Huang, D. & Kraus, W. L. The expanding universe of PARP1-mediated molecular and therapeutic mechanisms. *Mol Cell* **82**, 2315-2334 (2022).

Figure. S1.

Figure. S1.

**ENPP1 expression in human CTC and downregulated transcriptomic signature in a murine model of engrafted CTC-in**

**a.** Boxplot of ENPP1 expression levels in human CTC isolated from breast cancer tumor patients from the indicated datasets. FKPB: Fragments Per Kilobase Million. WBC: White blood cells.

**b.** Hierarchical functional GO categories obtained by transcriptomic analysis of gene signatures of 2 different independently isolated CTC-in cells derived from each ANV5 and 4T1 cell lines as compared to their respective parental cells evaluated by RNA-seq. Number of coherent genes with B>5 appear in parenthesis for each cell line. Selected categories are in a blue box.

**c.** Heatmap of downregulated genes in CTC-in derived from ANV5 and 4T1 cells assessed by RNA-seq of GO categories of “Radiation resistance” (*left panel*) and “Stemness” (*right panel*).

**d.** Immunoblots assessing the protein expression levels of a subset of genes in cells overexpressing ENPP1 (OE) compared to parental (C) cells.

Figure. S2.

Figure. S2.

**In vitro radiosensitivity, stability and post-translational modifications induced by ENPP1i**

**a.** Extracellular cGAMP measured after 24 h treatment with ENPP1i (AVA-NP-695) post-IR.

**b.** *Left panels:* Clonogenic assays with independently isolated CTC-in derived from ANV5 (803) and 4T1 (1592), CTC-in with silenced levels of ENPP1. Right inset depicts ENPP1 protein levels. *Right panels:* Cell viability in CTC-in with silenced levels for ENPP1 and Control CTC-in (shControl).

**c.** Cell growth kinetics by MTS in CTC-in cells.

**d.** Cell growth kinetics at day 5 of CTC-in cells (*Top panels*) or human cells (*Bottom panels*) treated with IR (2Gy), ENPP1i. ENPP1i was used at 2.72 µM for OE-ANV5 and 5 µM for OE-4T1 and their respective CTC-in derivatives.

**e.** Similar as in **d.** with the indicated cell lines. One-way ANOVA was used for comparison. *P<0.05; **P<0.01; ***P<0.001.

**f.** Relative gene expression levels of ATM, PARP1 and ENPP1 in CTC-in (*Left panels*) and OE cells (*Right panels*) derived from ANV5 and 4T1 cells treated with IR (2Gy) or IR and ENPP1i assessed by RT-qPCR.

**g.** Immunoblots of cell lysates of the indicated conditions incubated with Vehicle or Actinomycin D.

**h.** Immunoblots of PARylation levels assessed by an anti-PAR antibody in parental, OE and OE total cell lysates incubated with H_2_O_2_ and ENPP1i for the indicated duration.

**i.** Immunoblots showing phospho-ATM and ATM in parental (C) and OE nuclear cell lysates. Cells were incubated with H_2_O_2_ and ENPP1i for the indicated period.

Figure. S3.

Figure. S3.

**Effects of ENPP1i and DDRi on DNA damage repair and cell survival**

**a.** Percentage of cells in different cell cycle phases after 24 h incubation with ENPP1i (5 µM) or vehicle.

**b.** *Left panels:* Survival assay of cells incubated with ATMi (6.25 nM for OE-ANV5 and 25 nM for OE-4T1) and ENPP1i (2.72 µM for OE-ENPP1 and 5 µM for OE-4T1). *Right panels:* Annexin V was measured by flow cytometry, 24 h after incubation with the indicated treatments. **c.** *Top* *Left panels:* Survival assay after incubation with the indicated ENPP1i, ATMi and IR (2Gy) in murine cell lines. ENPP1i used in OE-ANV5 and OE-4T1 was 2.72 µM and 5 µM, whereas ATMi was used at 6.25 nM and 25 nM, respectively. *Top Right panels***:** Annexin V measured 24 h after incubation with the indicated treatments (ENPP1i: 2.72 µM and ATMi 5 µM). *Bottom panels:* Survival assay after incubation with the indicated ENPP1i, ATMi and IR alone or in combination in cell lines with high levels of ENPP1.

**d.** Survival assay upon incubation with ATMi (6.25 nM for OE-ANV5 and 25 nM for OE-4T1), DNA-Pks i(2.5 µM), PARPi (5 µM and 7.5 µM) and ENPP1i (2.72 µM and 5 µM) for OE-ENPP1 and OE-4T1 respectively. For MDA-MB-231, ATMi(2.5 µM) alone or in combination with ENPP1i (10 µM).

**e.** Survival assay of CTC-in cells and silenced with shRNAs targeting two different sequences (shENPP1a and b) treated with ATMi, PARPi and DNA-Pkcsi.

**f.** Evaluation of the percentage of positively labeled cells with an anti-γH2AX antibody performed by immunofluorescence and quantified by an in-house developed macro based on ImageJ®. Cells were incubated for the indicated duration with ENPP1i (5 µM) and ATMi (5 µM). n > 100 cells were examined. Mean and SD are represented. One-way ANOVA was used, followed by Dunnett´s multiple comparisons test against the Control group. *P<0.05, **P<0.01, ***P<0.001.

Figure. S4.

Figure. S4.

**Orthotopic tumor cell growth kinetics upon ENPP1i/DDRi post-IR treatment and the induction of STING signaling**

**a.** Representative images of H/E staining of sections obtained from the liver, heart and kidneys of naïve (non-tumor bearing), Control and triple-treated mice. Scale bar=100 µm

**b.** Tumor volume after orthotopic implantation of OE-4T1 cells treated with FD (6.2 Gy x 4) alone or in combination with ATMi (15 mg/kg daily), or with ATMi and ENPP1i (12 mg/kg daily, BID), or the triple combination (n= 5 mice per group). Kruskal-Wallis was used for comparison. Median and interquartile range is represented. ***P<0.0001.

**c.** Waterfall plot at the day of sacrifice.

**d.** Expression levels assessed by RT-qPCR of IFNα and IFNγ in OE-4T1 and OE-ANV5 cells treated as indicated.

**e.** Heatmap of selected cytokines with strong immunosuppressive activity overexpressed in OE-ANV5 as compared to parental ANV5 cells.

Figure. S5

Figure. S5

Gating strategy for different immune subpopulations

Figure. S6.

Figure. S6.

ENPP1 levels in human breast tumors by scRNA-seq

a. Gene expression levels of ENPP1 in the indicated cells subpopulations of human breast tumors assessed by scRNA-seq (*left panel*) and in different subset of CAFs (*right panel*).

b. UMAP of the tumor cell compartment showing the expression of the indicated genes upregulated in the engrafted CTC-in that tangentially overlap with ENPP1 expression levels.

c. Correlation of gene expression levels of ENPP1 with CRY1 and DDIT3 in human breast cancer tumors. Percentage of expression levels are represented.

d. UMAP of the expression of different genes in the subset of CAFs. UMAP of the fibroblast compartment yields eight supervised clusters of CAFs, one of which colocalizes with FAP and ITGB1 expression, and also superimposes with high ENPP1 expression levels. This subset identified as myCAFs (myofibroblast-type CAFs) also expresses the indicated genes.

Figure. S7.

Figure. S7.

Dosimetry in mice bearing single mammary tumor or bilateral mammary tumors

a. Representative dose distribution of focal irradiation in mice bearing on single mammary tumor. 3D-reconstruction of the tumor-bearing mouse with the irradiation beam represented as a blueish shadow. Isodose curves on a representative tumor-bearing mouse are shown in a CT-scan (axial, sagittal and coronal sections).

b. A dose volume histogram (DVH) represents in green line the treated tumor. The inset summary table provides relative doses in cGy (mean, minimal and maximal values) for treated tumor lesions.

c. Representative distribution of focal irradiation in mice bearing two mammary tumors in opposite inguinal mammary glands. 3D-reconstruction of the tumor-bearing mouse with the irradiated beam represented with a blueish shadow. Isodose curves on a representative tumor-bearing mouse are shown in a CT-scan (axial, sagittal and coronal sections) with the ipsilateral (to be irradiated right tumor in green) and the contralateral left tumor out of the irradiation field in yellow.

d. A dose volume histogram (DVH) represents in green line the treated tumor and in yellow line the non-irradiated tumor lesion. The inset summary table provides relative doses in cGy (mean, minimal and maximal values) for treated and non-treated tumor lesions.

Table S1.

**Histological evaluation of surgical resection margins of excised tumors**

Table S2.

**List of antibodies used for immunoblotting**

Table S3.

**List of antibodies used for immunophenotyping**

Table S4.

List of antibodies used for immunofluorescence

Table S5.

List of antibodies used for multiplexed tumor immunophenotyping

Table S6.

List of pharmacological inhibitors

Table S7.

List of target sequences for shRNA silencing

Table S8.

List of primer sequences for RT-qPCR
