# Supplementary material for: Dual ENPP1/ATM depletion blunts DNA damage repair boosting radioimmune efficacy to abrogate triple-negative breast cancer
Source: Signal Transduct Target Ther. 2025 Jun 13;10:185. doi: 10.1038/s41392-025-02271-2 (PMC12162836; doi:10.1038/s41392-025-02271-2)
Supplement: Supplementary file 2 — Data set containing uncropped Gels [file 41392_2025_2271_MOESM2_ESM.pdf]

Supplementary Materials for

Dual ENPP1/ATM depletion blunts DNA damage repair boosting radioimmune efficacy to abrogate triple-negative breast cancer

Borja Ruiz-Fernández de Córdoba§, Karmele Valencia§, Connor Welch, Haritz Moreno, Susana Martínez-Canarias, Carolina Zandueta, Eduardo Gómez, Alfonso Calvo, Nerea Otegui, Mirari Echepare, Ignacio Garzón, Daniel Ajona, David Lara-Astiaso, Elisabet Guruceaga, Laura Guembe, Rubén Pío, Ignacio Melero, Silvestre Vicent, Fernando Pastor, Rafael Martínez-Monge*, Fernando Lecanda*

Correspondence to: rmartinezm@unav.es and flecanda@unav.es

This PDF file includes:

Materials and Methods
Figures. S1 to S7
Tables S1 to S8

MATERIAL AND METHODS

Orthotopic injection

All experiments were performed in compliance with institutional guidelines approved by the Local Animal Ethics Committee (CEEA114-19) according to European Council Guidelines. All experiments were executed in female mice since sex did not represent a biological variable in this study. Mice were anesthetized by using isoflurane. Local shaving and disinfection were performed in the area surrounding the 4th inguinal mammary gland. A small longitudinal incision of 0.5–1 cm was performed 2 mm adjacent to the nipple. A total of 50 µl (25 µl Matrigel and 25 µl of Cell suspension in PBS) containing 2 x 10^4 ANV5 or 4T1 cells were then slowly delivered into the mammary gland of 6- to 7-week-old syngeneic FVB/N or Balb/C mice (Envigo, Barcelona, Spain), respectively, using a 0.3 ml insulin syringe with 29 G needle. The skin was sutured with absorbable surgical 6–0 silk (Vicryl). To evaluate orthotopic tumor growth kinetics, tumor size was measured manually with a digital caliper and tumor volume was calculated using the ellipsoid formula $V = \pi/6\ (L \times W \times H)$. Maximal tumor volume permitted was <1500 mm^3. To evaluate local tumor recurrence, when tumors reached the indicated volume, tumors were resected *en bloc* and immediately ink-marked and fixed for pathological evaluation. Surgical margin assessment is summarized in **Table S1**. Lung metastases were analyzed by H/E histological evaluation and quantification of metastatic area and total lung area and the number of metastatic nodules, by using ImageJ analysis.

Isolation of Circulating Tumor Cells (CTC)

Briefly, 4T1 cells were lentivirally transduced to express GFP for tracking and isolation. After intramammary inoculation in immunodeficient mice (Rag2tm1.1Flv Il2rgtm1.1Flv/J), tumors were resected at the appropriate time. Total blood was extracted under anesthesia, prior to sacrifice, and mixed with complete medium in p-150 plates. Tumor cells (CTC evading the tumor or CTC-out) were selected with puromycin. For ANV5, CTC-out could not be isolated because of the low numbers of CTC present in this model. To obtain CTC engrafting in the resected tumor bed or CTC-in, ANV5 parental cells or 4T1 CTC-out were inoculated in the left cardiac ventricle in 6-week-old female immune-compromised mice. Tumors were disaggregated to single-cell suspension, and after selection with 4-6 µg/mL of puromycin, CTC-in were cultured until single-cell derived colonies were detected.

In vivo depletion of immune subpopulations

In vivo depletion of CD8$^+$, CD4$^+$ T cells and natural killer (NK) cells was achieved by weekly intraperitoneal injections (100 µg per mouse, 3 times per week) of anti-mouse CD8α (clone YTS 169.4), anti-mouse CD4 (clone GK1.5), and anti-mouse NK1.1 (clone PK136) antibodies, respectively (Bio X Cell). Rat IgG2a isotype (clone 2A3) antibodies were used as control (Bio X Cell). Survival was monitored daily, and tumor volume was measured until maximum allowed size or at the end of the experimental period.

Fluorescent Traffic Light reporter (TLR) system

This system described elsewhere [1] includes a viral transduction system composed by I-SceI-T2A-IFP (endonuclease restriction enzyme), the Donor-T2A-BFP (Donor template) and the TLR (Reporter system). Briefly, TLR generates a readout for homology-directed repair (HR) mediated gene targeting and mutagenic non-homologous end joining (mutNHEJ) mediated gene disruption occurring at a designed individual DNA breakpoint. This fluorescent reporter is designed as such, as a double strand break (DSB) is produced at an embedded I-SceI nuclease cleavage site. The DSB repair generates distinct fluorescent signals upon resolution either through HR (where a functional green fluorescent protein eGFP open reading frame is

reestablished) with the aid of the exogenous donor template or through mutNHEJ (where non-repaired GFP DSB, places a mCherry coding sequence in frame to signal gene disruption). We transduced cells containing a single copy of the TLR integrated into the genome, transduced with I-SceI with or without the donor template transduction. After 72 h, cells were analyzed by flow cytometry. Cells acquiring the Donor template were sorted by V450-PB-A, whereas cells transduced with I-SceI were positive for R712-APC 700A. Cells transduced with TLR positive for I-SceI and Donor template were analyzed for Cherry (Y610-Cherry) and FITC (B525-FITC). The percentage of cells with FITC and Cherry were referenced to total cells (positive for V450-PB-A and R712-APC 700A).

Comet Assay
DNA damage was evaluated using the CometAssay® (#4250-050-K) (Biotechne, R&Dsystems) using the alkaline electrophoresis method. Cells were treated with IR, ENPP1i, or the combination for 24 h. Treated cells (500-1000) were seeded CometSlide™ according to the manufacturer's recommendations. Quantitative data were obtained using an automated macro developed in Fiji software (ImageJ®).

Immunofluorescence staining
Cells were seeded in 6-well plates containing coverslips. Following IR and adequate incubation time to allow for DDR, cells were fixed with 4% formaldehyde in PBS for 15 min, and permeabilized with 1%Triton X-100 PBS solution for 30 min. After blocking with 2% BSA for one hour, cells were washed for three times in PBS and subsequently incubated with anti-γH2AX antibody (Cell Signaling, 9718; 1:200) overnight. Next day, cells were incubated with anti-rabbit IgG-Alexa Fluor 555-conjugated antibodies (Invitrogen, A31572; 1:200) for 30 min to 1 h at room temperature and cells were stained with 100 ng/mL 4, 6-diamidino-2-phenylindole (DAPI, Biotium, 40009; 2,5 μg/ml) for one hour to visualize DNA. The cover slips were rinsed, mounted onto glass slides using antifade solution and sealed. Imaging was performed with a Zeiss Axiovert 200 microscope (Zeiss) equipped with a Perkin Elmer Spinning disk unit, a Hammamatsu C9100-50 CCD camera, and a 40× Plan Apochromat Objective (N.A. 0.95). Quantitative data were obtained using an automated macro developed in Fiji software (ImageJ®).

Preparation of Protein Lysates for PARylation Assay
Cell culture conditions were consistent with previously described methods. On day -1, 1,000,000 cells were plated in a 100 mm culture dish. Starting 24 hours later (day 0), the existing media was discarded, and cells were exposed to freshly prepared media containing the treatment agent (5 mM H2O2). Cells were incubated with H_2O_2 for periods ranging from 10 min to 2 h. At the end of the treatment, cells and supernatants were separated and centrifuged at 1,200 rpm for 5 minutes. The supernatant was then removed, and the cell pellets were lysed with lysis buffer as previously outlined[2], using RIPA buffer [1% Nonidet P-40, 50 mmol/L Tris-HCl (pH 7.4), and 150 mmol/L NaCl] enriched with protease inhibitors (Sigma) and 10 μM PJ34 (Thermo Fisher Scientific) to prevent PARP1 activation during lysate preparation

Radiation procedures
Cell DNA damage was induced by ionizing radiation (IR) with the indicated Gray (Gy) using a Nordion GammaCell 3000 irradiator. For non-invasive application of orthotopic tumors, animals were irradiated with a dual-focus-X-Ray tube external beam on a small animal radiation research platform (SARRP) from XStrahl, at 220kV and 13 mA, at the indicated doses (**Sup. Fig. 7**). In fractionated irradiation, 4 fractions of 6.2 Gy each, twice a day, at least 6 h apart, were delivered over two consecutive days. Single dose irradiation of 15 Gy and fractionated irradiation of 24.8 Gy (6.2 Gy x 4) are equi-effective as per the Linear-Quadratic formulation.

REFERENCES

1 Certo, M. T. *et al.* Tracking genome engineering outcome at individual DNA breakpoints. *Nature methods* **8**, 671-676 (2011).

2 Huang, D. & Kraus, W. L. The expanding universe of PARP1-mediated molecular and therapeutic mechanisms. *Mol Cell* **82**, 2315-2334 (2022).

Figure. S1.

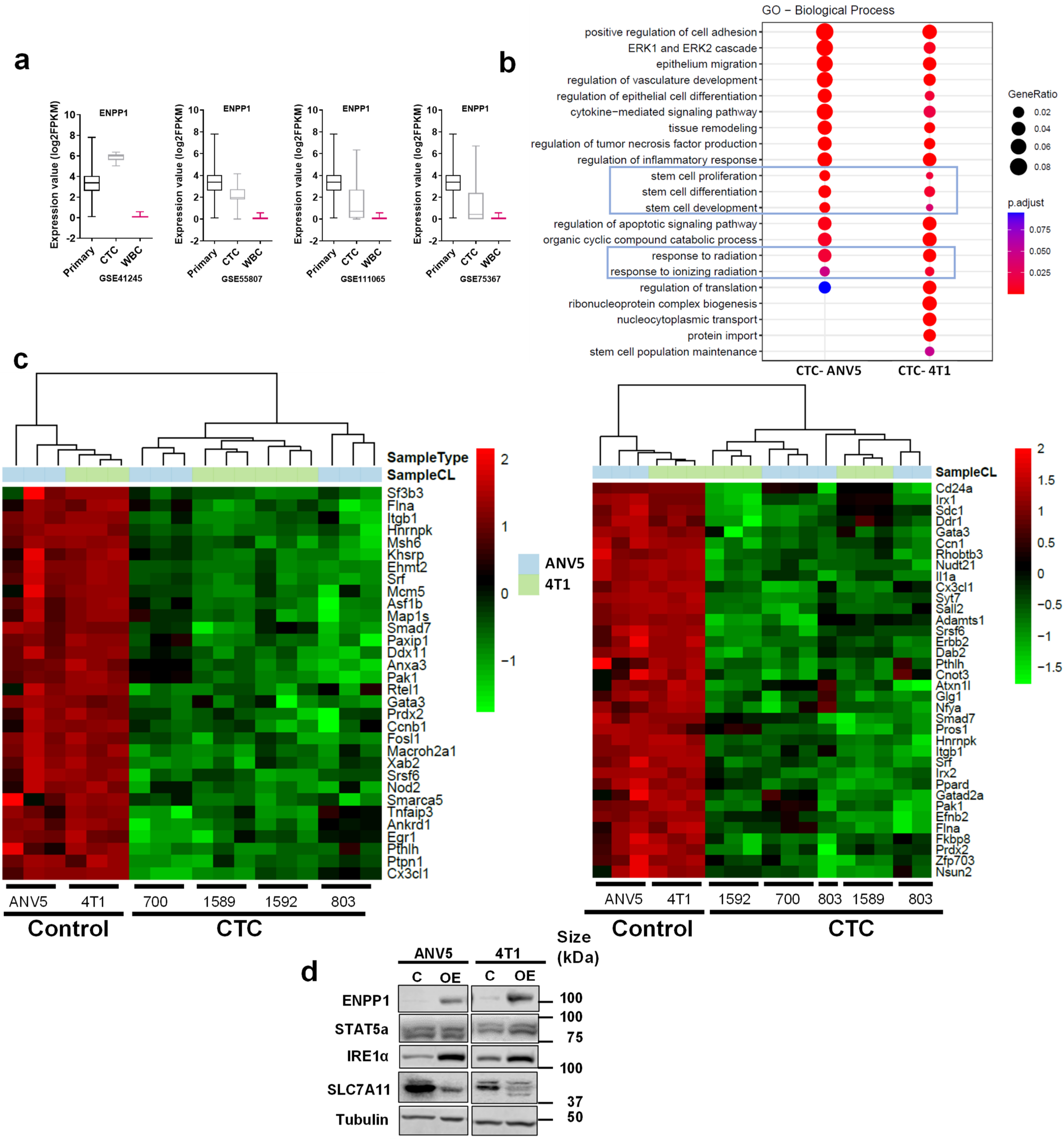

a
ENPP1
Expression value (log2FPKM)
Primary
CTC
WBC
GSE41245
GSE55807
GSE111065
GSE75367
b
GO – Biological Process
positive regulation of cell adhesion
ERK1 and ERK2 cascade
epithelium migration
regulation of vasculature development
regulation of epithelial cell differentiation
cytokine−mediated signaling pathway
tissue remodeling
regulation of tumor necrosis factor production
regulation of inflammatory response
stem cell proliferation
stem cell differentiation
stem cell development
regulation of apoptotic signaling pathway
organic cyclic compound catabolic process
response to radiation
response to ionizing radiation
regulation of translation
ribonucleoprotein complex biogenesis
nucleocytoplasmic transport
protein import
stem cell population maintenance
CTC- ANV5
CTC- 4T1
GeneRatio
0.02
0.04
0.06
0.08
p.adjust
0.075
0.050
0.025
c
SampleType
SampleCL
ANV5
4T1
Sf3b3
Flna
Itgb1
Hnrnpk
Msh6
Khsrp
Ehmt2
Srf
Mcm5
Asf1b
Map1s
Smad7
Paxip1
Ddx11
Anxa3
Pak1
Rtel1
Gata3
Prdx2
Ccnb1
Fosl1
Macroh2a1
Xab2
Srsf6
Nod2
Smarca5
Tnfaip3
Ankrd1
Egr1
Pthlh
Ptpn1
Cx3cl1
ANV5 4T1 700 1589 1592 803
Control
CTC
SampleCL
Cd24a
Irx1
Sdc1
Ddr1
Gata3
Ccn1
Rhobtb3
Nudt21
Il1a
Cx3cl1
Syt7
Sall2
Adamts1
Srsf6
Erbb2
Dab2
Pthlh
Cnot3
Atxn1l
Glg1
Nfya
Smad7
Pros1
Hnrnpk
Itgb1
Srf
Irx2
Ppard
Gatad2a
Pak1
Efnb2
Flna
Fkbp8
Prdx2
Zfp703
Nsun2
ANV5 4T1 1592 700 803 1589 803
Control
CTC
d
ANV5
4T1
C OE C OE
Size (kDa)
ENPP1
STAT5a
IRE1α
SLC7A11
Tubulin
100
100
75
100
37
50

Figure. S1.

ENPP1 expression in human CTC and downregulated transcriptomic signature in a murine model of engrafted CTC-in

a. Boxplot of ENPP1 expression levels in human CTC isolated from breast cancer tumor patients from the indicated datasets. FKPB: Fragments Per Kilobase Million. WBC: White blood cells.
b. Hierarchical functional GO categories obtained by transcriptomic analysis of gene signatures of 2 different independently isolated CTC-in cells derived from each ANV5 and 4T1 cell lines as compared to their respective parental cells evaluated by RNA-seq. Number of coherent genes with B>5 appear in parenthesis for each cell line. Selected categories are in a blue box.
c. Heatmap of downregulated genes in CTC-in derived from ANV5 and 4T1 cells assessed by RNA-seq of GO categories of "Radiation resistance" (*left panel*) and "Stemness" (*right panel*).
d. Immunoblots assessing the protein expression levels of a subset of genes in cells overexpressing ENPP1 (OE) compared to parental (C) cells.

Figure. S2.

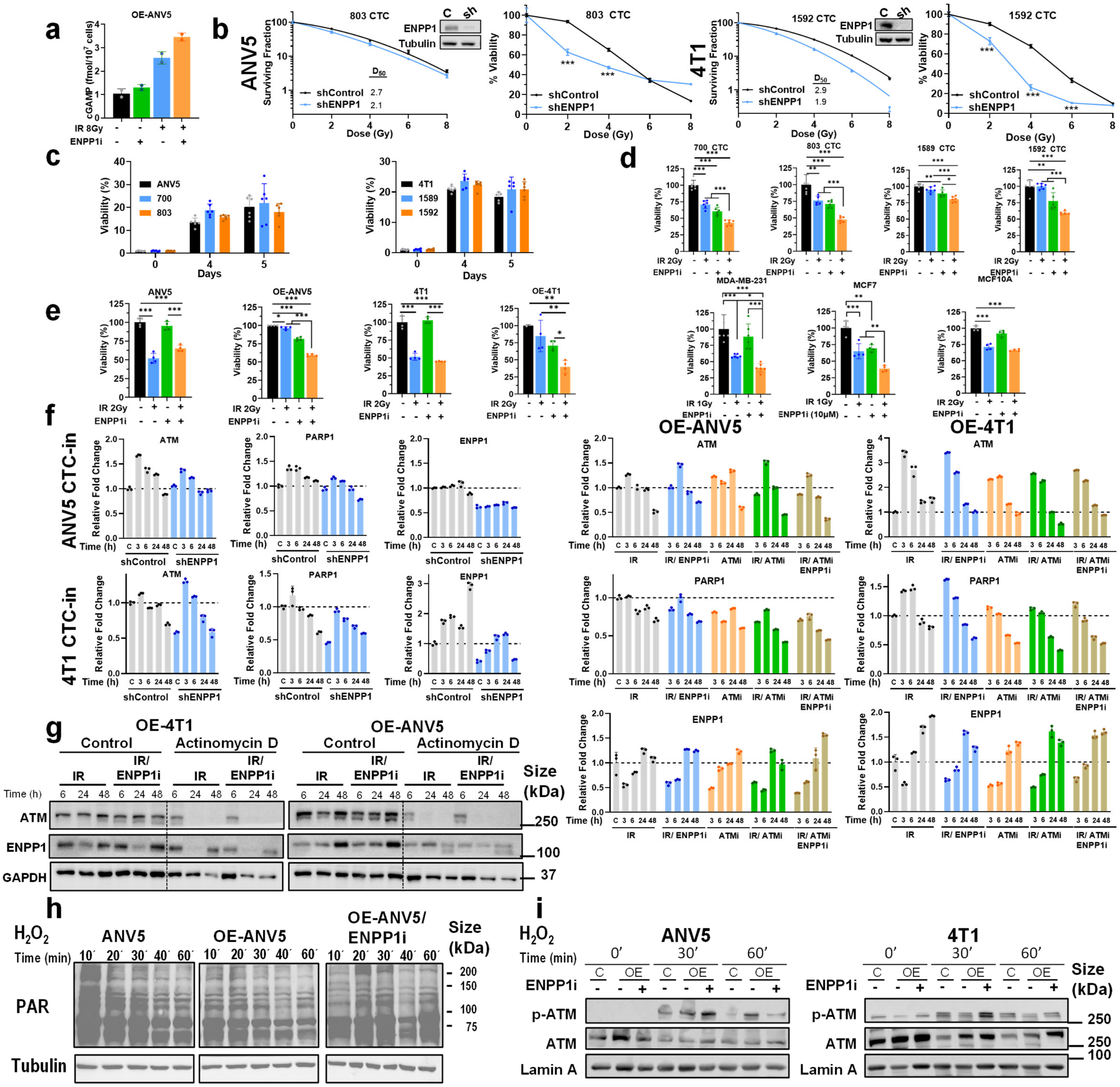
a
b
c
d
e
f
g
h
i
OE-ANV5
ANV5
4T1
803 CTC
1592 CTC
shControl
shENPP1
Dose (Gy)
Surviving Fraction
% Viability
Viability (%)
Days
OE-4T1
ANV5 CTC-in
4T1 CTC-in
ATM
PARP1
ENPP1
Relative Fold Change
Control
Actinomycin D
IR
IR/ ENPP1i
ATMi
IR/ ATMi
Size (kDa)
GAPDH
PAR
Tubulin
p-ATM
Lamin A
ENPP1i
Time (min)
Time (h)

Figure. S2.

In vitro radiosensitivity, stability and post-translational modifications induced by ENPP1i

a. Extracellular cGAMP measured after 24 h treatment with ENPP1i (AVA-NP-695) post-IR.

b. *Left panels:* Clonogenic assays with independently isolated CTC-in derived from ANV5 (803) and 4T1 (1592), CTC-in with silenced levels of ENPP1. Right inset depicts ENPP1 protein levels. *Right panels:* Cell viability in CTC-in with silenced levels for ENPP1 and Control CTC-in (shControl).

c. Cell growth kinetics by MTS in CTC-in cells.

d. Cell growth kinetics at day 5 of CTC-in cells (*Top panels*) or human cells (*Bottom panels*) treated with IR (2Gy), ENPP1i. ENPP1i was used at 2.72 µM for OE-ANV5 and 5 µM for OE-4T1 and their respective CTC-in derivatives.

e. Similar as in **d.** with the indicated cell lines. One-way ANOVA was used for comparison. *P<0.05; **P<0.01; ***P<0.001.

f. Relative gene expression levels of ATM, PARP1 and ENPP1 in CTC-in (*Left panels*) and OE cells (*Right panels*) derived from ANV5 and 4T1 cells treated with IR (2Gy) or IR and ENPP1i assessed by RT-qPCR.

g. Immunoblots of cell lysates of the indicated conditions incubated with Vehicle or Actinomycin D.

h. Immunoblots of PARylation levels assessed by an anti-PAR antibody in parental, OE and OE total cell lysates incubated with H_2O_2 and ENPP1i for the indicated duration.

i. Immunoblots showing phospho-ATM and ATM in parental (C) and OE nuclear cell lysates. Cells were incubated with H_2O_2 and ENPP1i for the indicated period.

Figure. S3.

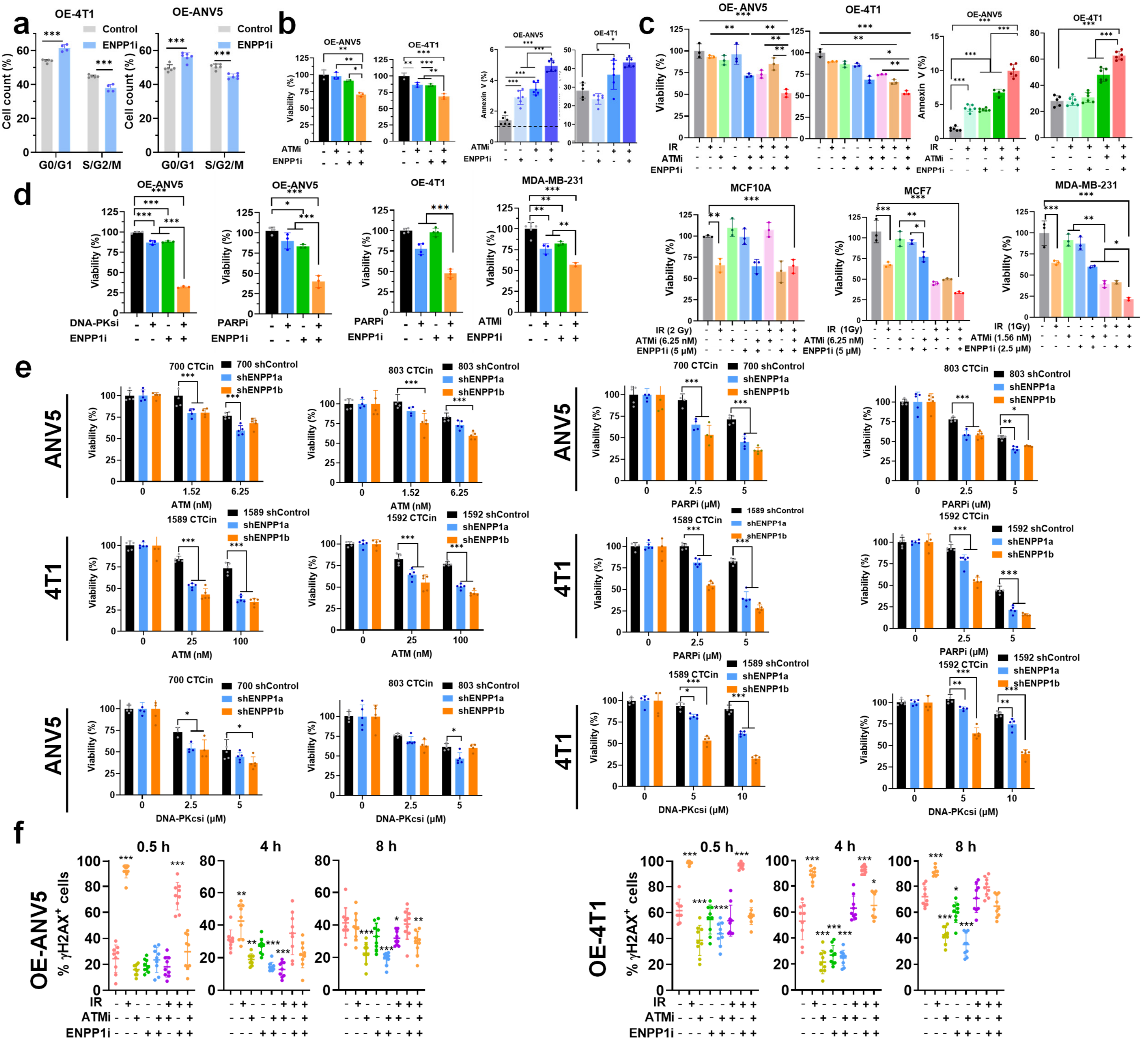

Figure. S3.

Effects of ENPP1i and DDRi on DNA damage repair and cell survival

a. Percentage of cells in different cell cycle phases after 24 h incubation with ENPP1i (5 µM) or vehicle.

b. *Left panels:* Survival assay of cells incubated with ATMi (6.25 nM for OE-ANV5 and 25 nM for OE-4T1) and ENPP1i (2.72 µM for OE-ENPP1 and 5 µM for OE-4T1). *Right panels:* Annexin V was measured by flow cytometry, 24 h after incubation with the indicated treatments. **c.** *Top Left panels:* Survival assay after incubation with the indicated ENPP1i, ATMi and IR (2Gy) in murine cell lines. ENPP1i used in OE-ANV5 and OE-4T1 was 2.72 µM and 5 µM, whereas ATMi was used at 6.25 nM and 25 nM, respectively. *Top Right panels*: Annexin V measured 24 h after incubation with the indicated treatments (ENPP1i: 2.72 µM and ATMi 5 µM). *Bottom panels:* Survival assay after incubation with the indicated ENPP1i, ATMi and IR alone or in combination in cell lines with high levels of ENPP1.

d. Survival assay upon incubation with ATMi (6.25 nM for OE-ANV5 and 25 nM for OE-4T1), DNA-Pks i(2.5 µM), PARPi (5 µM and 7.5 µM) and ENPP1i (2.72 µM and 5 µM) for OE-ENPP1 and OE-4T1 respectively. For MDA-MB-231, ATMi(2.5 µM) alone or in combination with ENPP1i (10 µM).

e. Survival assay of CTC-in cells and silenced with shRNAs targeting two different sequences (shENPP1a and b) treated with ATMi, PARPi and DNA-Pkcsi.

f. Evaluation of the percentage of positively labeled cells with an anti-γH2AX antibody performed by immunofluorescence and quantified by an in-house developed macro based on ImageJ®. Cells were incubated for the indicated duration with ENPP1i (5 µM) and ATMi (5 µM). n > 100 cells were examined. Mean and SD are represented. One-way ANOVA was used, followed by Dunnett´s multiple comparisons test against the Control group. *P<0.05, **P<0.01, ***P<0.001.

Figure. S4.

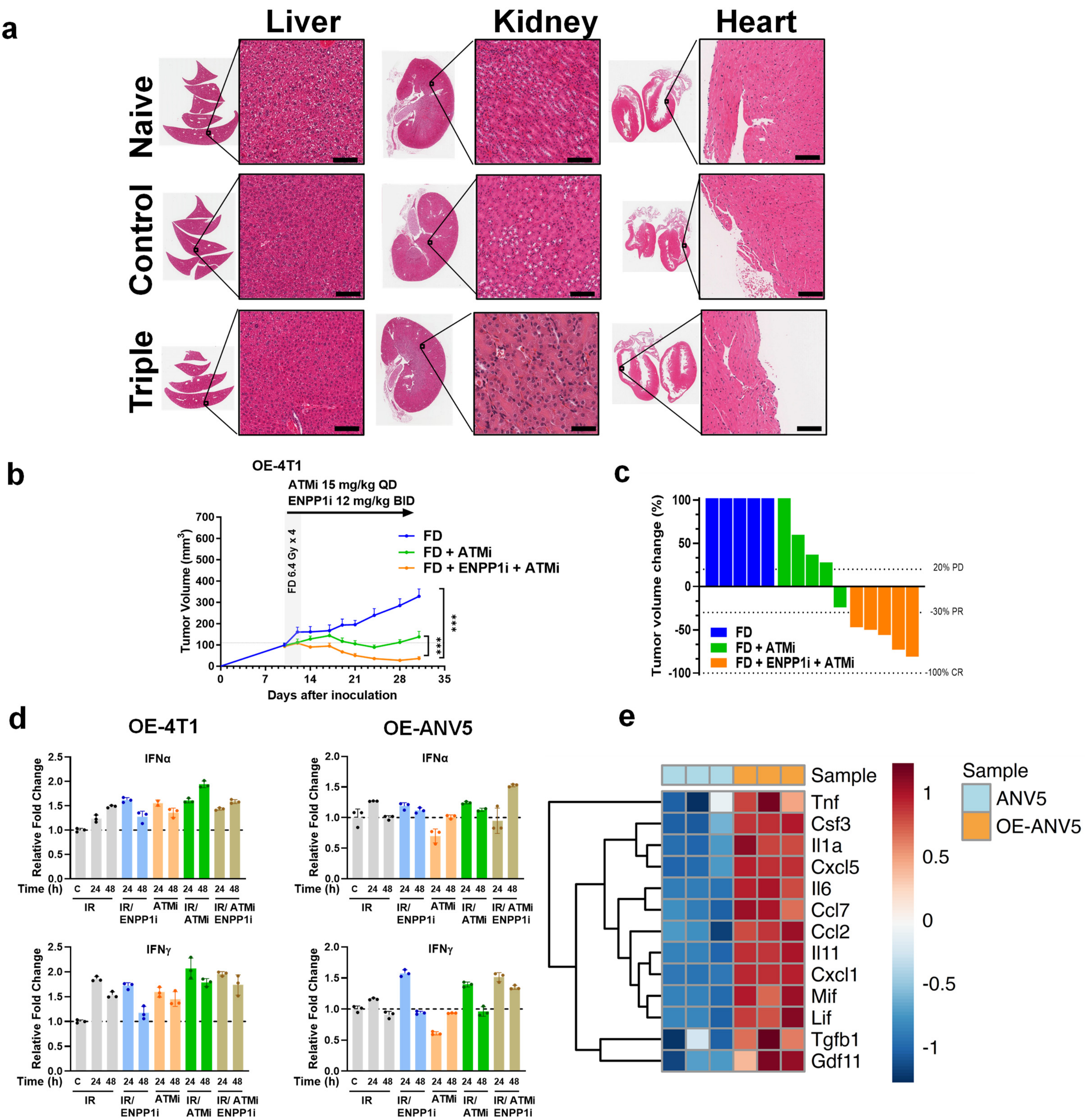
a
Liver
Kidney
Heart
Naive
Control
Triple
b
OE-4T1
ATMi 15 mg/kg QD
ENPP1i 12 mg/kg BID
FD 6.4 Gy x 4
Tumor Volume (mm³)
FD
FD + ATMi
FD + ENPP1i + ATMi
***
***
Days after inoculation
c
Tumor volume change (%)
20% PD
-30% PR
-100% CR
FD
FD + ATMi
FD + ENPP1i + ATMi
d
OE-4T1
OE-ANV5
IFNα
IFNγ
Relative Fold Change
Time (h)
C 24 48 24 48 24 48 24 48 24 48
IR
IR/ ENPP1i
ATMi
IR/ ATMi
IR/ ATMi ENPP1i
e
Sample
Tnf
Csf3
Il1a
Cxcl5
Il6
Ccl7
Ccl2
Il11
Cxcl1
Mif
Lif
Tgfb1
Gdf11
ANV5
OE-ANV5

Figure. S4.

Orthotopic tumor cell growth kinetics upon ENPP1i/DDRi post-IR treatment and the induction of STING signaling

a. Representative images of H/E staining of sections obtained from the liver, heart and kidneys of naïve (non-tumor bearing), Control and triple-treated mice. Scale bar=100 μm
b. Tumor volume after orthotopic implantation of OE-4T1 cells treated with FD (6.2 Gy x 4) alone or in combination with ATMi (15 mg/kg daily), or with ATMi and ENPP1i (12 mg/kg daily, BID), or the triple combination (n= 5 mice per group). Kruskal-Wallis was used for comparison. Median and interquartile range is represented. ***P<0.0001.
c. Waterfall plot at the day of sacrifice.
d. Expression levels assessed by RT-qPCR of IFNα and IFNγ in OE-4T1 and OE-ANV5 cells treated as indicated.
e. Heatmap of selected cytokines with strong immunosuppressive activity overexpressed in OE-ANV5 as compared to parental ANV5 cells.

Figure. S5

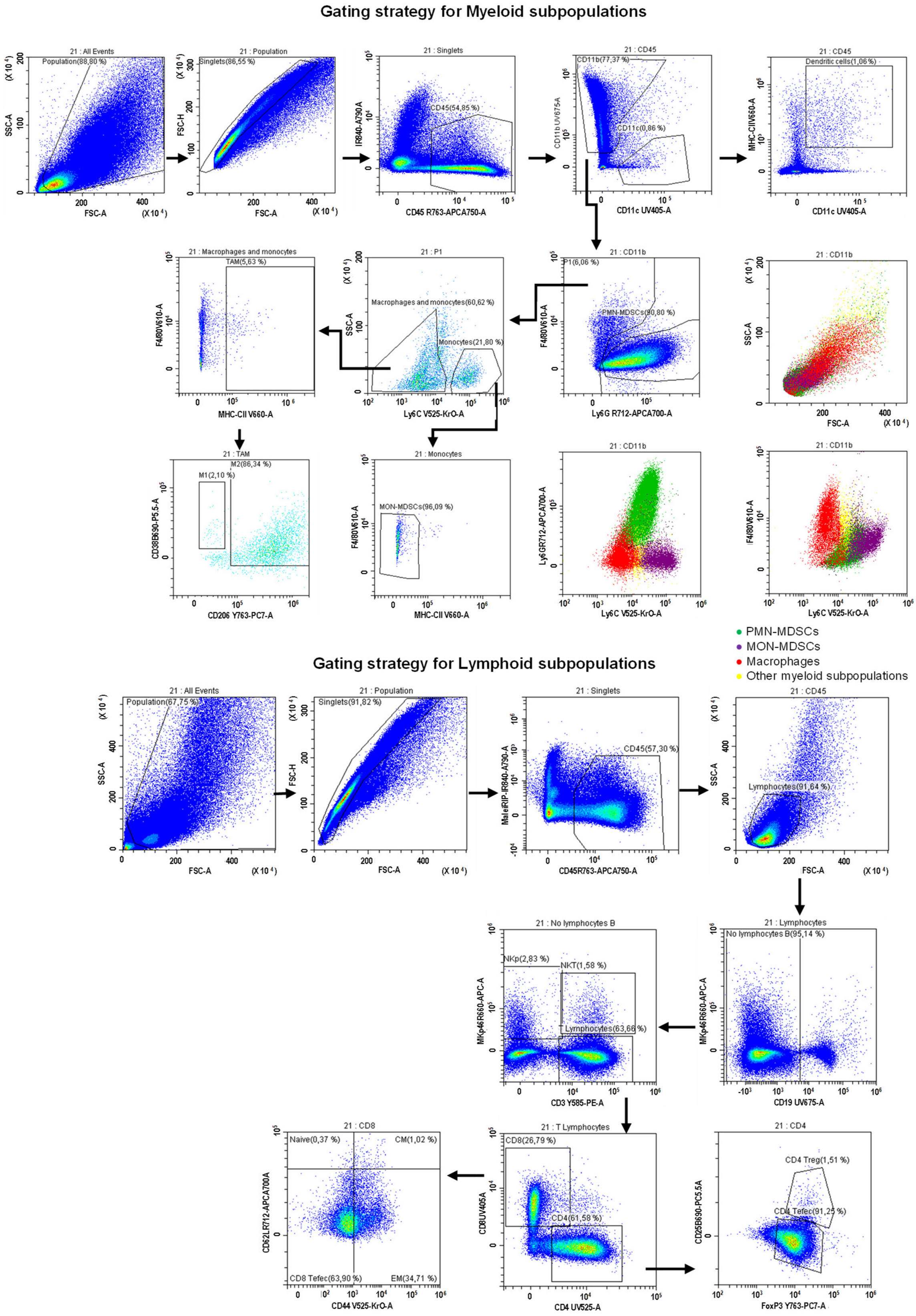

Gating strategy for Myeloid subpopulations
21 : All Events
Population(88,80 %)
SSC-A
FSC-A
21 : Population
Singlets(86,55 %)
FSC-H
21 : Singlets
CD45(54,85 %)
IR840-A790A
CD45 R763-APCA750-A
21 : CD45
CD11b(77,37 %)
CD11c(0,86 %)
CD11b UV675A
CD11c UV405-A
21 : CD45
Dendritic cells(1,06 %)
MHC-CIIV660-A
CD11c UV405-A
21 : Macrophages and monocytes
TAM(5,63 %)
F4/80V610-A
MHC-CII V660-A
21 : P1
Macrophages and monocytes(60,62 %)
Monocytes(21,80 %)
SSC-A
Ly6C V525-KrO-A
21 : CD11b
P1(6,06 %)
PMN-MDSCs(80,80 %)
F4/80V610-A
Ly6G R712-APCA700-A
21 : CD11b
SSC-A
FSC-A
21 : TAM
M2(86,34 %)
M1(2,10 %)
CD38B690-P5.5-A
CD206 Y763-PC7-A
21 : Monocytes
MON-MDSCs(96,09 %)
F4/80V610-A
MHC-CII V660-A
21 : CD11b
Ly6GR712-APCA700-A
Ly6C V525-KrO-A
21 : CD11b
F4/80V610-A
Ly6C V525-KrO-A
PMN-MDSCs
MON-MDSCs
Macrophages
Other myeloid subpopulations
Gating strategy for Lymphoid subpopulations
21 : All Events
Population(67,75 %)
SSC-A
FSC-A
21 : Population
Singlets(91,82 %)
FSC-H
FSC-A
21 : Singlets
CD45(57,30 %)
MaleRIP-IR840-A790-A
CD45R763-APCA750-A
21 : CD45
Lymphocytes(91,64 %)
SSC-A
FSC-A
21 : No lymphocytes B
NKp(2,83 %)
NKT(1,58 %)
T Lymphocytes(63,66 %)
MKp46R660-APC-A
CD3 Y585-PE-A
21 : Lymphocytes
No lymphocytes B(95,14 %)
MKp46R660-APC-A
CD19 UV675-A
21 : CD8
Naive(0,37 %)
CM(1,02 %)
CD8 Tefec(63,90 %)
EM(34,71 %)
CD62LR712-APCA700A
CD44 V525-KrO-A
21 : T Lymphocytes
CD8(26,79 %)
CD4(61,58 %)
CD8UV405A
CD4 UV525-A
21 : CD4
CD4 Treg(1,51 %)
CD4 Tefec(91,25 %)
CD25B690-PC5.5A
FoxP3 Y763-PC7-A

Figure. S5
Gating strategy for different immune subpopulations

Figure. S6.

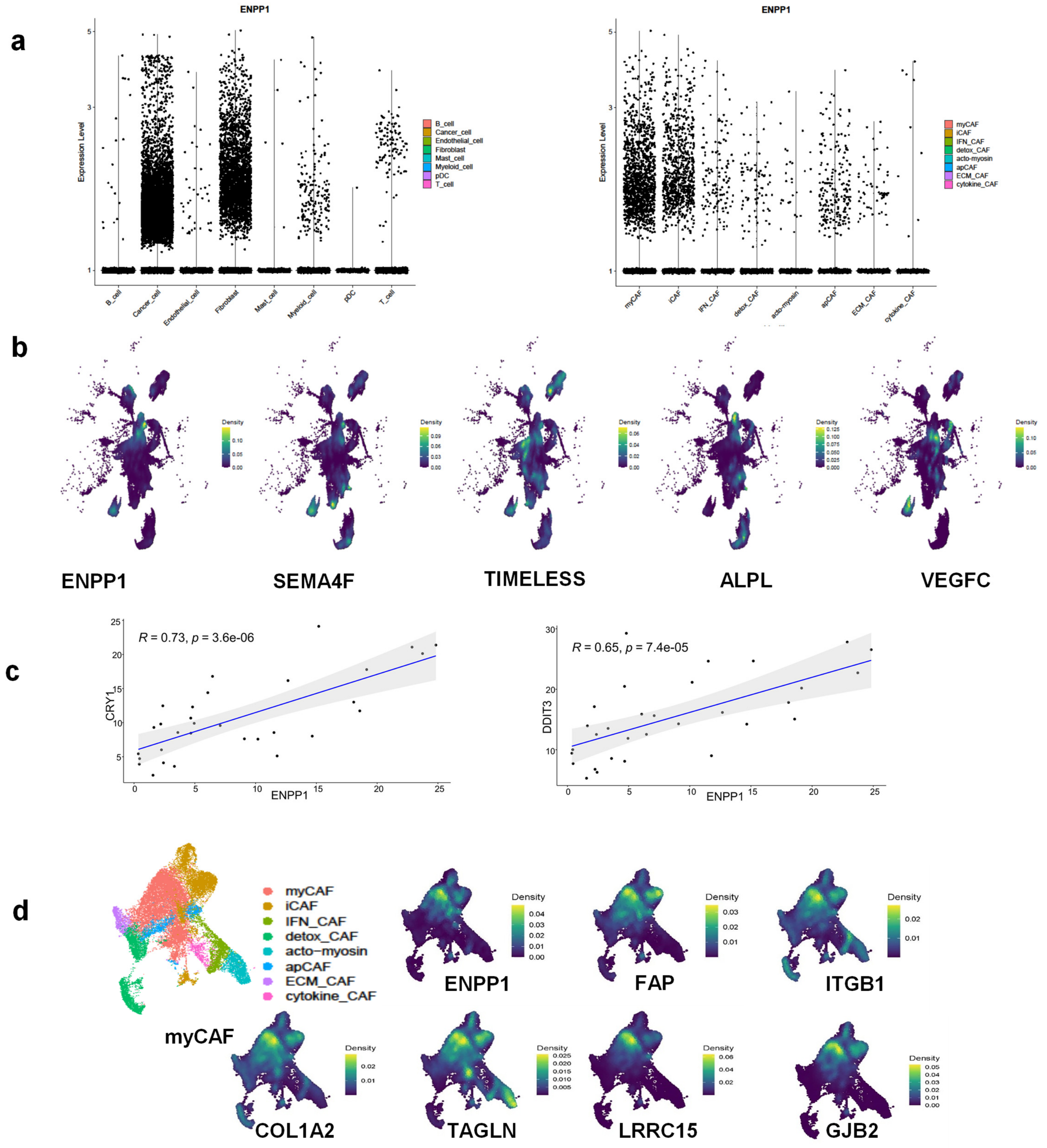
a
ENPP1
Expression Level
B_cell
Cancer_cell
Endothelial_cell
Fibroblast
Mast_cell
Myeloid_cell
pDC
T_cell
ENPP1
myCAF
iCAF
IFN_CAF
detox_CAF
acto-myosin
apCAF
ECM_CAF
cytokine_CAF
b
ENPP1
SEMA4F
TIMELESS
ALPL
VEGFC
Density
c
R = 0.73, p = 3.6e-06
CRY1
ENPP1
R = 0.65, p = 7.4e-05
DDIT3
ENPP1
d
myCAF
iCAF
IFN_CAF
detox_CAF
acto−myosin
apCAF
ECM_CAF
cytokine_CAF
myCAF
ENPP1
FAP
ITGB1
COL1A2
TAGLN
LRRC15
GJB2
Density

Figure. S6.
ENPP1 levels in human breast tumors by scRNA-seq

a. Gene expression levels of ENPP1 in the indicated cells subpopulations of human breast tumors assessed by scRNA-seq (*left panel*) and in different subset of CAFs (*right panel*).
b. UMAP of the tumor cell compartment showing the expression of the indicated genes upregulated in the engrafted CTC-in that tangentially overlap with ENPP1 expression levels.
c. Correlation of gene expression levels of ENPP1 with CRY1 and DDIT3 in human breast cancer tumors. Percentage of expression levels are represented.
d. UMAP of the expression of different genes in the subset of CAFs. UMAP of the fibroblast compartment yields eight supervised clusters of CAFs, one of which colocalizes with FAP and ITGB1 expression, and also superimposes with high ENPP1 expression levels. This subset identified as myCAFs (myofibroblast-type CAFs) also expresses the indicated genes.

Figure. S7.

a

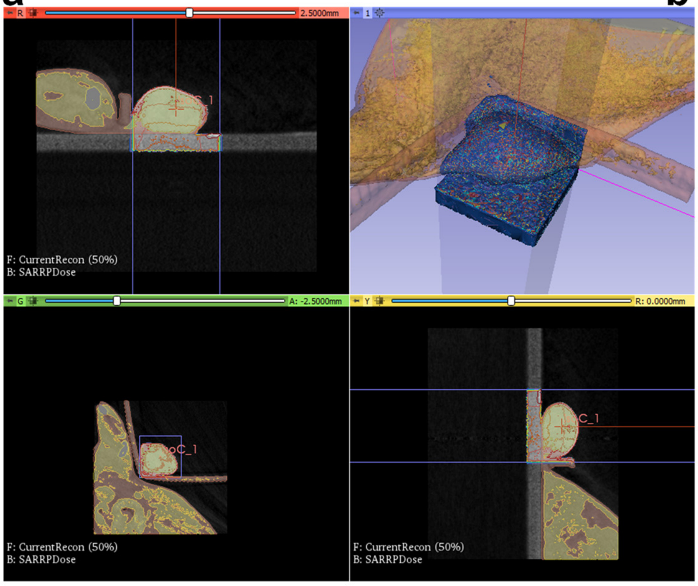

b

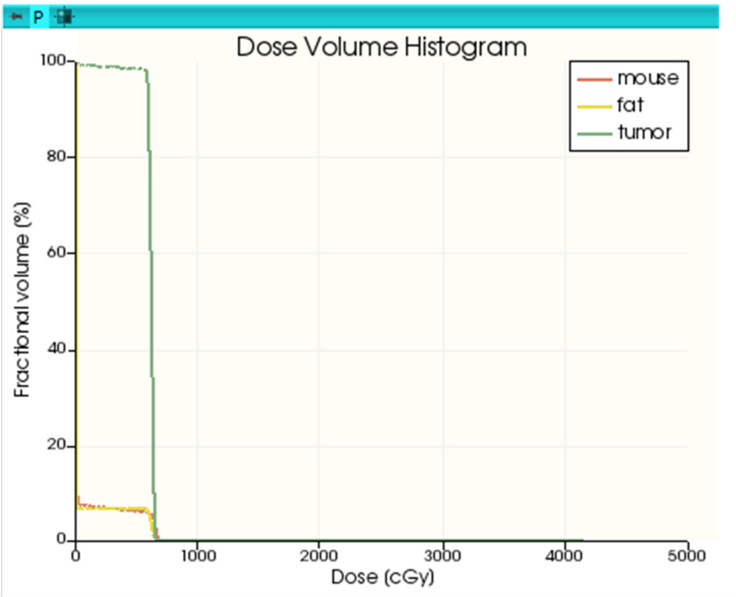

Structure	Volume (mm3)	Mean dose (cGy)	Min dose (cGy)	Max dose (cGy)
Mouse	164659	47.0071	0.00413526	687.176
Fat	233543	44.4507	0.0366124	669.486
Tumor	158.37	609.728	8.71352	669.486

c

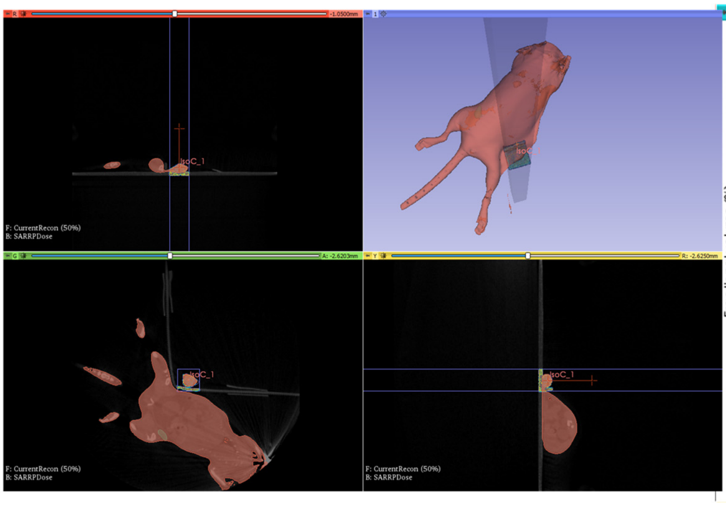

d

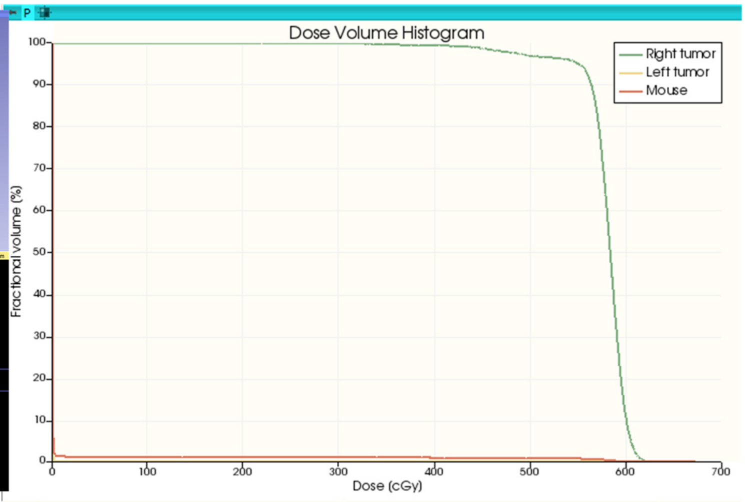

Structure	Volume (mm³)	Mean dose (cGy)	Min dose (cGy)	Max dose (cGy)
Irradiated (Right) tumor	56.1234	578.645	132.432	630.793
Contrateral (Left) tumor	31.3416	0.16056	0	1.96668
Mouse	174865	5.26681	0	633.602

Figure. S7.
Dosimetry in mice bearing single mammary tumor or bilateral mammary tumors

a. Representative dose distribution of focal irradiation in mice bearing on single mammary tumor. 3D-reconstruction of the tumor-bearing mouse with the irradiation beam represented as a blueish shadow. Isodose curves on a representative tumor-bearing mouse are shown in a CT-scan (axial, sagittal and coronal sections).
b. A dose volume histogram (DVH) represents in green line the treated tumor. The inset summary table provides relative doses in cGy (mean, minimal and maximal values) for treated tumor lesions.
c. Representative distribution of focal irradiation in mice bearing two mammary tumors in opposite inguinal mammary glands. 3D-reconstruction of the tumor-bearing mouse with the irradiated beam represented with a blueish shadow. Isodose curves on a representative tumor-bearing mouse are shown in a CT-scan (axial, sagittal and coronal sections) with the ipsilateral (to be irradiated right tumor in green) and the contralateral left tumor out of the irradiation field in yellow.
d. A dose volume histogram (DVH) represents in green line the treated tumor and in yellow line the non-irradiated tumor lesion. The inset summary table provides relative doses in cGy (mean, minimal and maximal values) for treated and non-treated tumor lesions.

Table S1.
Histological evaluation of surgical resection margins of excised tumors

Fig Ref	Groups	R0	R1	R2	χ^2
**4h**	Control	1	7	7	ns
	ENPP1i	0	4	11	
	ATMi	1	8	6	
	ENPP1i + ATMi	2	5	8	
**4i**	FD	3	6	5	ns
	FD + ATMi	5	6	3	
	FD +ATMi +ENPP1i	4	6	5	

ns: non-significant

Table S2.
List of antibodies used for immunoblotting

Target	Organism	Host	Reference	Provider	Dilution	Weight (KDa)	Activity
Caspase-3	Human, Mouse, Rat, Monkey	Rabbit	9662S	Cell Signalling	1:1000	17, 19, 35 KDa	WB IP IHC
cGAS	Mouse	Rabbit	31659T	Cell Signalling	1:1000	62 KDa	WB IP
ENPP1	Human, Mouse, Rat	Rabbit	40036	Cell Signalling	1:1000	110 KDa	WB
GAPDH	Human, Mouse, Rat, Monkey	Rabbit	5174	Cell Signalling	1:5000	37 KDa	WB IHC IF F
PARP	Human, Mouse, Rat, Monkey	Rabbit	9542	Cell Signalling	1:1000	89, 116 KDa	WB
P-ATM	Human, Mouse, Rat	Mouse	MA1-2020	Invitrogen	1:1000	370 KDa	WB IP IF
ATM	Human, Mouse, Rat	Rabbit	ab201022	Abcam	1:1000	370 KDa	WB IP IF Chip F
γH2AX (Ser139)	Human, Mouse, Rat, Monkey	Rabbit	9718	Cell Signalling	1:1000	15 KDa	WB IHC IF F
p-TBK1	Human, Mouse	Rabbit	5483	Cell Signalling	1:1000	84 KDa	WB IP IF F
TBK1	Human, Mouse, Rat, Monkey	Rabbit	3504	Cell Signalling	1:1000	84 KDa	WB IP
Poly/Mono-ADP Ribose (D9P7Z)	All	Rabbit	89190	Cell Signalling	1:1000	15-250 KDa	WB IF F
IRE1α (14C10)	Human, Mouse, Rat	Rabbit	3294	Cell Signalling	1:1000	130 Kda	WB IP
Stat5a (D2O6Y)	Human, Mouse, Rat	Rabbit	94205	Cell Signalling	1:1000	90 KDa	WB IP ChIP
RAD51 (D4B10)	Human, Mouse, Rat, Monkey	Rabbit	8875	Cell Signalling	1:1000	37 KDa	WB IP
Tubulin	Human, Mouse	Mouse	T4026	Sigma-Aldrich	1:5000	50 KDa	WB
Vinculin	Human, Mouse, Rat, Monkey	Rabbit	13901T	Cell Signalling	1:1000	124 KDa	WB IHC F

Table S3.
List of antibodies used for immunophenotyping

Channel	Antibody	Clone	Company	Reference	Dilution
PE	CD3e	(20/70)	BioLegend	553063	1:200
APC	αCD200R3	(Ba13)	BioLegend	142208	1:40
APC	NKp46 (CD335)	(29A1.4)	BioLegend	137608	1:20
APC-H7	αCD45	(30-F11)	BioLegend	103116	1:500
FITC	αCD193	(J073E5)	BioLegend	144510	1:80
FITC	αGITR	(DTA-1)	BioLegend	126308	1:100
AF700	αLy6G	(1A8)	BioLegend	127622	1:200
AF700	αCD62L	(MEL-14)	BioLegend	104426	1:800
PerCP/Cy5,5	αCD38	90	BioLegend	102722	1:160
PerCP/Cy5,5	αCD25	(PC61)	BioLegend	102028	1:50
PECy7	αCD206	(C068C2)	BioLegend	141720	1:40
PECy7	αFoxP3	(3G3)	Abcam	ab210232	1:320
BV421	αPD-L1	(10F.9G2)	BioLegend	124315	1:80
BV510	αLy6C	(HK1.4)	BioLegend	128033	1:200
BV510	αCD44	(IM7)	BioLegend	103044	1:200
BV605	αF4/80	(BM8)	BioLegend	123133	1:80
BV605	αPD-1	(29F.1A12)	BioLegend	135220	1:80
BV650	αMHC-C2	(M5/114.15.2)	BioLegend	107641	1:80
BUV395	αCD11c	(HL3)	BD Bioscience	564080	1:80
BUV395	αCD8	(53-6.7)	BD Bioscience	563786	1:200
BUV496	CD4	(GK1.5)	BD Bioscience	564667	1:400
BUV661	CD11b	(M1/70)	BD Bioscience	565080	1:160
BUV661	CD19	(1D3)	BD Bioscience	565076	1:200
Purified Rat Anti-Mouse CD16/CD32 (Mouse BD Fc Block™)		Clone 2.4G2	BD Bioscience	553142	1:200
MaleiMide			Promocell	PK-PF840-3-5	
eBioscience™ Permeabilization Buffer (10X)			Invitrogen	00-8333-56	
eBioscience™ Fixation/Permeabilization Diluent			EBIOSCIENCE	00-5223-56	
eBioscience™ Fixation/Permeabilization Concentrate			EBIOSCIENCE	00-5123-43	

Table S4.
List of antibodies used for immunofluorescence

Antibody	Reference	Dilution	Provider
Purified Rat Anti-Mouse CD16/CD32 (Mouse BD Fc Block™)	553142	1:200	BD Bioscience
Alexa Fluor® 488 anti-mouse CD31	102514	1:200	BioLegend
PE/Cyanine7 anti-mouse CD326 (Ep-CAM)	118215	1:200	BioLegend
Brilliant Violet 421™ anti-mouse CD140A (PDGFRα)	562774	1:400	BD Bioscience
Brilliant Violet 510™ anti-mouse CD45	103138	1:400	BioLegend
PE/Cyanine7 anti-mouse CD45	103114	1:400	BioLegend
Brilliant Violet 510™ anti-mouse Ly-6G	127633	1:200	BioLegend
PE anti-mouse/human CD11b	101208	1:200	BioLegend
Brilliant Violet 421™ anti-mouse F4/80	123132	1:400	BioLegend
Alexa Fluor® 488 anti-mouse CD11c	117311	1:200	BioLegend
PerCP/Cyanine5.5 anti-mouse I-A/I-E	107626	1:400	BioLegend
APC-H7 Zombie NIR™ Fixable Viability	423106	1:1000	BioLegend

Table S5.
List of antibodies used for multiplexed tumor immunophenotyping

Antibodies (Ref)	Dilution	Antigen retrieval	Opal
FOXP3 (CST, D608R)	1:500	Citrate buffer, pH6	480
PD-1 (CST, D7D5W)	1:100	EDTA buffer, pH9	690
CD4 (Abcam ab183685)	1:400	Citrate buffer, pH6	520
CD8a (CST, D4W2Z)	1:500	Citrate buffer, pH6	620
F4/80 (CST, D259R)	1:400	Citrate buffer, pH6	570
CD86 (CST, ESW6H)	1:300	Citrate buffer, pH6	690
Arg1 (CST, D4E3M)	1:200	EDTA buffer, pH9	620

Table S6.
List of pharmacological inhibitors

Name	Target	Reference	Provider
Olaparib	PARPi	HY-10162	MedChemExpress
RI-1	RAD51i	HY-15317	MedChemExpress
Ceralasertib	ATRi	HY-19323	MedChemExpress
AZD-1390	ATMi	HY-109566	MedChemExpress
AZD-7648	DNA-PKsi	HY-111783	MedChemExpress
ML216	BLMi	HY-12342	MedChemExpress
Mirin	MRNi	HY-117693	MedChemExpress
CX-5461	RNA-Polli	HY-13323	MedChemExpress
Palbociclib	CDK4/6i	HY-50767	MedChemExpress
AZD-7762	CHK1/2i	HY-10992	MedChemExpress
B12536	PLKi	HY-50698	MedChemExpress
AVA-NP-695	ENPP1i	-	AVAMMUNE
Actinomycin D	RNASEi	A1410	Sigma-Aldrich
H_2O_2 33% w/v	DNA	141077.1211	Panreac

Table S7.
List of target sequences for shRNA silencing

Gene	TRCN		Sequence (5'->3')
**ENPP1**	**shRNAa TRCN0000436447**	**Fw**	**5'-CCGGCCGATTTGGGTGACCGCTAATCTCGAGATTAGCGGTCACCCAAATCGGTTTTTG-3'**
		**Rv**	**5'-AATTCAAAAACCGATTTGGGTGACCGCTAATCTCGAGATTAGCGGTCACCCAAATCGG-3'**
	**shRNAb TRCN0000080635**	**Fw**	**5'-CCGGCCAGAGACATACTATTCATTTCTCGAGAAATGAATAGTATGTCTCTGGTTTTTG-3'**
		**Rv**	**5'-AATTCAAAAACCAGAGACATACTATTCATTTCTCGAGAAATGAATAGTATGTCTCTGG-3'**
**TIMELESS**	**shRNA TRCN0000234048**	**Fw**	**5'-CCGGTGGGTCCGTCTCGTAGCAAATCTCGAGATTTGCTACGAGACGGACCCATTTTTG-3'**
		**Rv**	**5'-AATTCAAAAATGGGTCCGTCTCGTAGCAAATCTCGAGATTTGCTACGAGACGGACCCA-3'**
	shRNA TRCN0000234045	Fw	5'-CCGGTGATCAGGCTGATGGTAAATTCTCGAGAATTTACCATCAGCCTGATCATTTTTG-3'
		Rv	5'-AATTCAAAAATGATCAGGCTGATGGTAAATTCTCGAGAATTTACCATCAGCCTGATCA-3'
	shRNA TRCN0000234047	Fw	5'-CCGGAGATACCATCCACGCTCATTCCTCGAGGAATGAGCGTGGATGGTATCTTTTTTG-3'
		Rv	5'-AATTCAAAAAAGATACCATCCACGCTCATTCCTCGAGGAATGAGCGTGGATGGTATCT-3'
**STAT5A**	**shRNA TRCN0000231566**	**Fw**	**5'-CCGGGCCATTCACGACGCGAGATTTCTCGAGAAATCTCGCGTCGTGAATGGCTTTTTG-3'**
		**Rv**	**5'-AATTCAAAAAGCCATTCACGACGCGAGATTTCTCGAGAAATCTCGCGTCGTGAATGGC-3'**
	shRNA TRCN0000231567	Fw	5'-CCGGCGAGGTCTTTGCCAAGTATTACTCGAGTAATACTTGGCAAAGACCTCGTTTTTG-3'
		Rv	5'-AATTCAAAAACGAGGTCTTTGCCAAGTATTACTCGAGTAATACTTGGCAAAGACCTCG-3'
	shRNA TRCN0000257383	Fw	5'-CCGGGACGTGAGATTCAAGTCTAACCTCGAGGTTAGACTTGAATCTCACGTCTTTTTG-3'
		Rv	5'-AATTCAAAAAGACGTGAGATTCAAGTCTAACCTCGAGGTTAGACTTGAATCTCACGTC-3'
**ERN1**	shRNA TRCN0000356311	Fw	5'-CCGGGGAATCCTCTACATGGGTAAACTCGAGTTTACCCATGTAGAGGATTCCTTTTTG-3'
		Rv	5'-AATTCAAAAAGGAATCCTCTACATGGGTAAACTCGAGTTTACCCATGTAGAGGATTCC-3'
	**shRNA TRCN0000232026**	**Fw**	**5'-CCGGCCAGCACAGTGGCCTAAATAGCTCGAGCTATTTAGGCCACTGTGCTGGTTTTTG-3'**
		**Rv**	**5'-AATTCAAAAACCAGCACAGTGGCCTAAATAGCTCGAGCTATTTAGGCCACTGTGCTGG-3'**
	shRNA TRCN0000232025	Fw	5'-CCGGCAGACAGATCTGCGCAAATTCCTCGAGGAATTTGCGCAGATCTGTCTGTTTTTG-3'
		Rv	5'-AATTCAAAAACAGACAGATCTGCGCAAATTCCTCGAGGAATTTGCGCAGATCTGTCTG-3'
**cGAS**	shRNA TRCN0000416658	Fw	5'-CCGGAGGAAATCCGCTGAGTCATTTCTCGAGAAATGACTCAGCGGATTTCCTTTTTTG-3'
		Rv	5'-AATTCAAAAAAGGAAATCCGCTGAGTCATTTCTCGAGAAATGACTCAGCGGATTTCCT-3'
	shRNA TRCN0000216225	Fw	5'-CCGGCTATTCTCTCAAGAACTAATTCTCGAGAATTAGTTCTTGAGAGAATAGTTTTTG-3'
		Rv	5'-AATTCAAAAACTATTCTCTCAAGAACTAATTCTCGAGAATTAGTTCTTGAGAGAATAG-3'
	**shRNA TRCN0000215476**	**Fw**	**5'-CCGGGAGATTGAAACGCAAAGATATCTCGAGATATCTTTGCGTTTCAATCTCTTTTTG-3'**
		**Rv**	**5'-AATTCAAAAAGAGATTGAAACGCAAAGATATCTCGAGATATCTTTGCGTTTCAATCTC-3'**

Table S8.
List of primer sequences for RT-qPCR

Gene	Organism	Name	Sequence (5'->3')
TIMELESS	Mouse	mTimeless Fw	GAGGCTTCAGAGGACTCGC
		mTimeless Rev	CAGAGCGGGCAGTAGAGATG
Ern1	Mouse	mErn1 Fw	CAACCTGCCCAAACATCGAG
		mErn1 Rev	GGTGGTCGGTGTGTTGTCT
HPRT	mouse	mHPRT Fw	TCCTCCTCAGACCGCTTTT
		mHPRT Rv	CCTGGTTCATCATCGCTAATC
HPRT	Mouse	mENPP1 FWD especif	GAGCTCGAGTCACCAGCCG
		mENPP1 RV especif	TAGGTGTTGGGGTCTTTGGCA
Cry1	Mouse	mCry1 FWD	CCGAGCTGTAGCGGTGGAA
		mCry1 RV	CGCGGAGCTTCTCCCTTG
Ddit3	Mouse	mDdit3 FWD	CCTGAGGAGAGAGTGTTCCAG
		mDdit3 Rv	CAAGGTGAAAGGCAGGGACT
SLC7A11	Mouse	mSlc7a11 FWD	ATGCATATGCTGGCTGGTTT
		mSlc7a11 Rv	AGATTGCAAGGGGGATGGTT
H2bc4	Mouse	mH2bc4 Fw	ACCAAGTACACCAGCTCCAAG
		mH2bc4 Rev	GTTACAGCATCCAGCACTGT
Icam1	Mouse	mIcam1 FWD	GCTTTGAGAACTGTGGCACC
		mIcam1 Rv	TGAGGTCCTTGCCTACTTGC
Gpnmb	Mouse	mGpnmb FWD	GTGTTCCCCAGATGCCAGAA
		mGpnmb RV	CCCCTGCAGTCCAGTTGTAG
Sem4f	Mouse	mSema4f FWD	CTTCGCTTTAACCCTGCCCT
		mSema4f Rv	AATTGTGACATTCGTCCTCTTTCT
Vegfc	Mouse	mVegfc FWD	GACGGGGGCGAGGTCAAG
		mVegfc Rv	TCAGCTCATCTACGCTGGACAC
Selp1	Mouse	mSelp1 FWD	TGGCTCTGCTAAGAAAGCGT
		mSelp1 Rv	GTTCCTAGGTGGCTGTGAGG
Cd24a	Mouse	mCd24a FWD	TTCTGGCACTGCTCCTACCC
		mCd24a RV	CTGGTTACCGGGAAACGGT
CD44	Mouse	mCD44 FWD	GGATCCGAATTAGCTGGACACT
		mCD44 RV	TGCCAGGAGAGATGCCAAGA
cGAS	Mouse	mcGASFw	GGAGCAAAATGCTGCAGAAAAG
		mcGASRv	TGCATCCAGCTCTTGAAACTCT
STAT5	Mouse	mSTAT5Fw	AGTCTCAGTTCAGCGTTGGC
		mSTAT5Rv	CGATAACGACCACAGGGAGG
PARP1	Mouse	mPARP1Fw	AAGGTGGGAAGGTGTTCAGC
		mPARP1Rv	CCGGAAGATCCAGTACCTGC
ATM	Mouse	mATMFw	GTGTGATTTTTCAGGGGATTTGG
		mATMRv	AGCAAACGTTGCCTGAATGAC
